# A Developmental Gene Expression Atlas Reveals Novel Biological Basis of Complex Phenotypes in Sheep

**DOI:** 10.1093/gpbjnl/qzaf020

**Published:** 2025-03-04

**Authors:** Bingru Zhao, Hanpeng Luo, Xuefeng Fu, Guoming Zhang, Emily L Clark, Feng Wang, Brian Paul Dalrymple, V Hutton Oddy, Philip E Vercoe, Cuiling Wu, George E Liu, Cong-jun Li, Ruidong Xiang, Kechuan Tian, Yanli Zhang, Lingzhao Fang

**Affiliations:** Jiangsu Livestock Embryo Engineering Laboratory, College of Animal Science and Technology, Nanjing Agricultural University, Nanjing 210095, China; Department of Immunology, School of Basic Medical Sciences, Capital Medical University, Beijing 100069, China; Key Laboratory of Genetics Breeding and Reproduction of Xinjiang Wool-sheep Cashmere-goat (XJYS1105), Institute of Animal Science, Xinjiang Academy of Animal Sciences, Urumqi 830011, China; Jiangsu Livestock Embryo Engineering Laboratory, College of Animal Science and Technology, Nanjing Agricultural University, Nanjing 210095, China; College of Veterinary Medicine, Nanjing Agricultural University, Nanjing 210095, China; The Roslin Institute, University of Edinburgh, Midlothian, EH25 9RG, United Kingdom; Jiangsu Livestock Embryo Engineering Laboratory, College of Animal Science and Technology, Nanjing Agricultural University, Nanjing 210095, China; CSIRO Agriculture, St Lucia, QLD 4067, Australia; School of Agriculture and Environment and Institute of Agriculture, The University of Western Australia, Perth, WA 6009, Australia; NSW Department of Primary Industries, Livestock Industries Centre, University of New England, Armidale, NSW 2351, Australia; School of Agriculture and Environment and Institute of Agriculture, The University of Western Australia, Perth, WA 6009, Australia; Key Laboratory of Special Environment Biodiversity Application and Regulation in Xinjiang / International Center for the Collaborative Management of Cross-border Pest in Central Asia College of Life Sciences, School of Life Sciences, Xinjiang Normal University, Urumqi 830053, China; Animal Genomics and Improvement Laboratory, Henry A. Wallace Beltsville Agricultural Research Center, Agricultural Research Service, USDA, Beltsville, MD 20705, USA; Animal Genomics and Improvement Laboratory, Henry A. Wallace Beltsville Agricultural Research Center, Agricultural Research Service, USDA, Beltsville, MD 20705, USA; Faculty of Veterinary & Agricultural Science, The University of Melbourne, Parkville, VIC 3052, Australia; Agriculture Victoria, AgriBio, Centre for AgriBiosciences, Bundoora, VIC 3083, Australia; Cambridge-Baker Systems Genomics Initiative, Baker Heart and Diabetes Institute, Melbourne, VIC 3004, Australia; CSIRO Agriculture, St Lucia, QLD 4067, Australia; Institue of Animal Science and Veterinary Medicine, Shandong Academy of Agricultural Sciences, Jinan 250032, China; Jiangsu Livestock Embryo Engineering Laboratory, College of Animal Science and Technology, Nanjing Agricultural University, Nanjing 210095, China; Center for Quantitative Genetics and Genomics (QGG), Aarhus University, 8000 Aarhus, Denmark

**Keywords:** Sheep, Developmental biology, Gene Expression Atlas, Genome-wide association study, Complex trait

## Abstract

Sheep (*Ovis aries*) represent one of the most important livestock species for global animal protein and wool production. However, little is known about the genetic and biological basis of ovine phenotypes, particularly those with high economic value and environmental impact. Here, by integrating 1413 RNA sequencing (RNA-seq) samples from 51 distinct tissues across 14 developmental time points, representing early-prenatal, late-prenatal, neonatal, lamb, juvenile, adult, and elderly stages, we constructed a high-resolution Developmental Gene Expression Atlas (dGEA) in sheep. We observed dynamic patterns of gene expression and regulatory networks across tissues and developmental stages. Leveraging this resource to interpret genetic associations for 48 monogenic and 12 complex traits in sheep, we found that genes upregulated at prenatal developmental stages played more important roles in shaping these phenotypes than those upregulated at postnatal stages. For instance, genetic associations of crimp number, mean staple length (MSL), and individual birthweight were significantly enriched in the prenatal rather than postnatal skin and immune tissues. By comprehensively integrating genome-wide association study (GWAS) fine-mapping results with the sheep dGEA, we identified several candidate genes for complex traits in sheep, such as *SOX9* for MSL, *GNRHR* for litter size at birth, and *PRKDC* for live weight. These results provide novel insights into the developmental and molecular architecture of ovine phenotypes. The dGEA (https://sheepdgea.njau.edu.cn/) will serve as an invaluable resource for sheep developmental biology, genetics, genomics, and selective breeding.

## Graphical abstract

**Figure:**
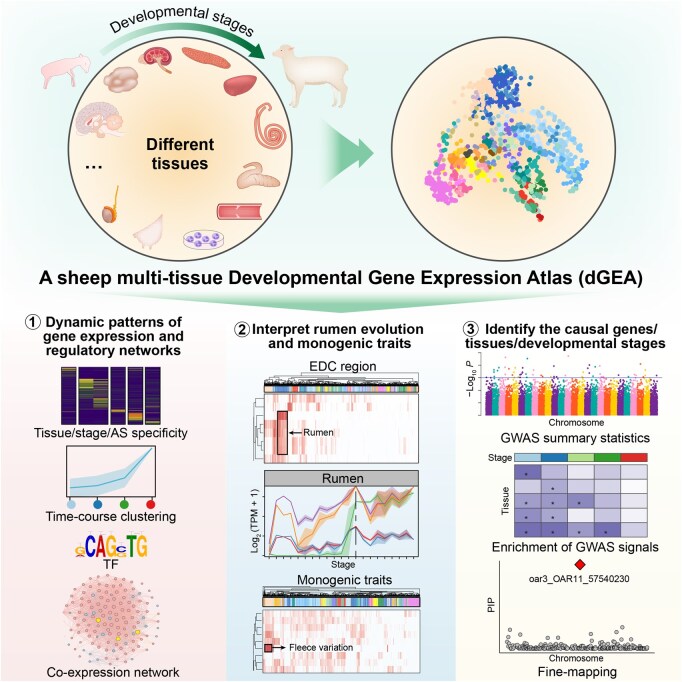

## Teaser

**Highlights**
 

1) A high-resolution developmental Gene Expression Atlas (dGEA) in sheep.
2) Dynamic gene expression patterns and regulatory networks across sheep development stages.
3) Prenatal gene expression has a stronger influence on sheep traits than postnatal stages.
4) Integration of GWAS and dGEA highlights candidate genes for complex traits in sheep.

## Introduction

Sheep (*Ovis aries*) represent one of the most important domestic animals for meat, milk, and wool production worldwide [1]. To meet the increasing demand for safe animal production while minimizing the associated negative environmental impacts, a sustainable genetic improvement program is urgently needed in sheep, balancing production, reproduction, health, and feed efficiency. A better understanding of the genetic and biological basis of these complex traits will accelerate their current genetic improvement *via* genomic selection and catalyze advanced precision breeding (*e.g.*, genome editing-based) and cell-based food systems in sheep [2–5].

Genome-wide association studies (GWAS) have uncovered many loci contributing to a wide range of complex traits in sheep [6]. For instance, the Animal QTLdb (August 25, 2024) curated 4743 quantitative trait loci (QTLs) from 270 publications, representing 217 traits, in sheep [7]. However, the causal genes/variants and molecular mechanisms (*e.g.*, tissues or cell types in which those genomic variants act) underlying most of those complex traits are largely unknown, partially due to (1) high linkage disequilibrium (LD) between causal variants and nearby markers, and (2) the lack of detailed functional annotations of these QTL regions.

Characterization of multi-tissue gene expression could offer valuable insights into the genetic and biological basis of complex traits [8,9]. In the past decades, great efforts have been made by the scientific community to functionally annotate genes in many species, such as the ENCODE [10] and Genotype-Tissue Expression (GTEx) projects in humans [11–15], and similar initiatives have existed for domestic animals including the Functional Annotation of Animal Genomes (FAANG) [15–18] and Farm Animal Genotype-Tissue Expression (FarmGTEx) project [19,20]. Since the reference genome was published in 2014 [1], several gene expression studies have also been conducted to improve the functional annotation of genes in sheep [4,21–23]. However, most of these studies are limited to either tissue types or developmental stages [21,24–27]. A systematic characterization of multi-tissue developmental transcriptomes will give us a great opportunity to unravel tissues and developmental stages in which trait-associated variants act, providing novel molecular mechanisms underpinning complex traits [28,29].

**Figure 1:**
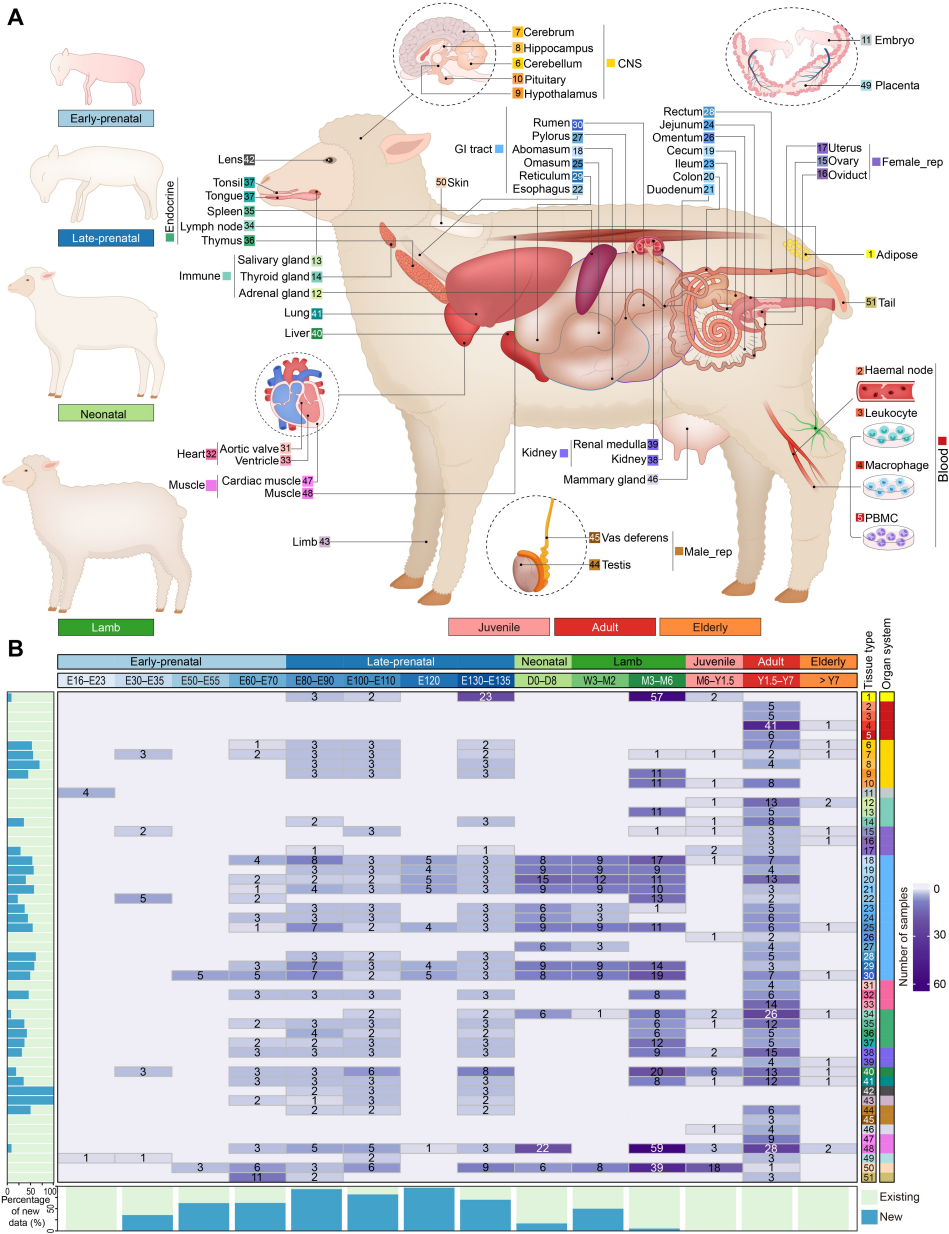
Data summary of the sheep multi-tissue dGEA **A**. Illustration of 51 different tissues across 7 developmental stages in the sheep dGEA, where the color coding for tissues and developmental stages is consistently used in all the figures throughout the entire manuscript. **B**. Distribution of samples from each tissue across various developmental stages. We classified 51 tissues into 20 organ systems (meta-tissue) and 14 time points into 7 developmental stages, *i.e.*, early-prenatal (≤ E70), late-prenatal (> E70), neonatal (D0–D8), lamb (D8–M6), juvenile (M6–Y1.5), adult (Y1.5–Y7), and elderly (> Y7). The value in the heatmap represents the sample size of a tissue at a developmental stage. The bar charts on the left and bottom represent the proportion of newly generated RNA-seq data in this study across tissues and developmental stages, respectively. dGEA, Developmental Gene Expression Atlas; CNS, central nervous system; GI, gastrointestinal; Female_rep, female reproductive; Male_rep, male reproductive; PBMC, peripheral blood mononuclear cell; E, embryonic day; D, days postnatal; W, weeks postnatal; M, months postnatal; Y, years postnatal.

Here, we constructed a Developmental Gene Expression Atlas (dGEA) in sheep by integrating 410 newly generated RNA sequencing (RNA-seq) samples (30 tissues across 10 developmental time points from 44 animals) and 1003 publicly available high-quality RNA-seq samples. The dGEA encompasses a total of 51 distinct tissue/cell types (hereafter referred to as “tissues”) originating from all three germ layers (endoderm, mesoderm, and ectoderm) across 14 developmental time points from embryonic day 15 (E15) to 7 years postnatal (Y7) (**Figure 1**A). Based on the known developmental biology in sheep [21,30], we classified these 14 time points into 7 stages, including early-prenatal (≤ E70), late-prenatal (> E70), neonatal (D0–D8; D refers to days postnatal), lamb (D8–M6; M refers to months postnatal), juvenile (M6–Y1.5), adult (Y1.5–Y7), and elderly (> Y7) (Figure 1B). We systematically investigated tissue- and development-specific features of the sheep transcriptome, and then utilized these biological insights to explore the development- and tissue-specific regulation of 48 monogenic and 12 complex traits with GWAS data (*n* = 1639) in sheep, including wool production, growth, and reproduction. Our results, for the first time, systemically establish connections among developmental stage, tissue, and phenotype in sheep. The dGEA (https://sheepdgea.njau.edu.cn/) will serve as a valuable resource for developmental biology, genetics, genomics, and selective breeding in sheep.

## Results

### Data summary in the sheep dGEA

A total of ∼ 45.5 billion clean reads were yielded with an averaged uniquely mapped rate of 81.79% across all the 1557 RNA-seq samples (Figure S1; Table S1). After filtering out low-quality samples and non-expressed genes [transcripts per million (TPM) < 0.1, details in Materials and methods], a total of 1413 RNA-seq samples and 26,423 genes were retained for subsequent analyses (Figure 1B, Figure S1). The number of expressed genes increased with the number of clean reads across samples and tissues (Figure S2A and B). Among different types of genes [*e.g.*, long intergenic non-coding RNAs (lncRNAs) and microRNAs (miRNAs)], protein-coding genes (PCGs) showed the lowest tissue specificity (TAU value) (Figure S2C), and many of them were ubiquitously expressed across all the developmental stages (Figure S2D–G).

**Figure 2:**
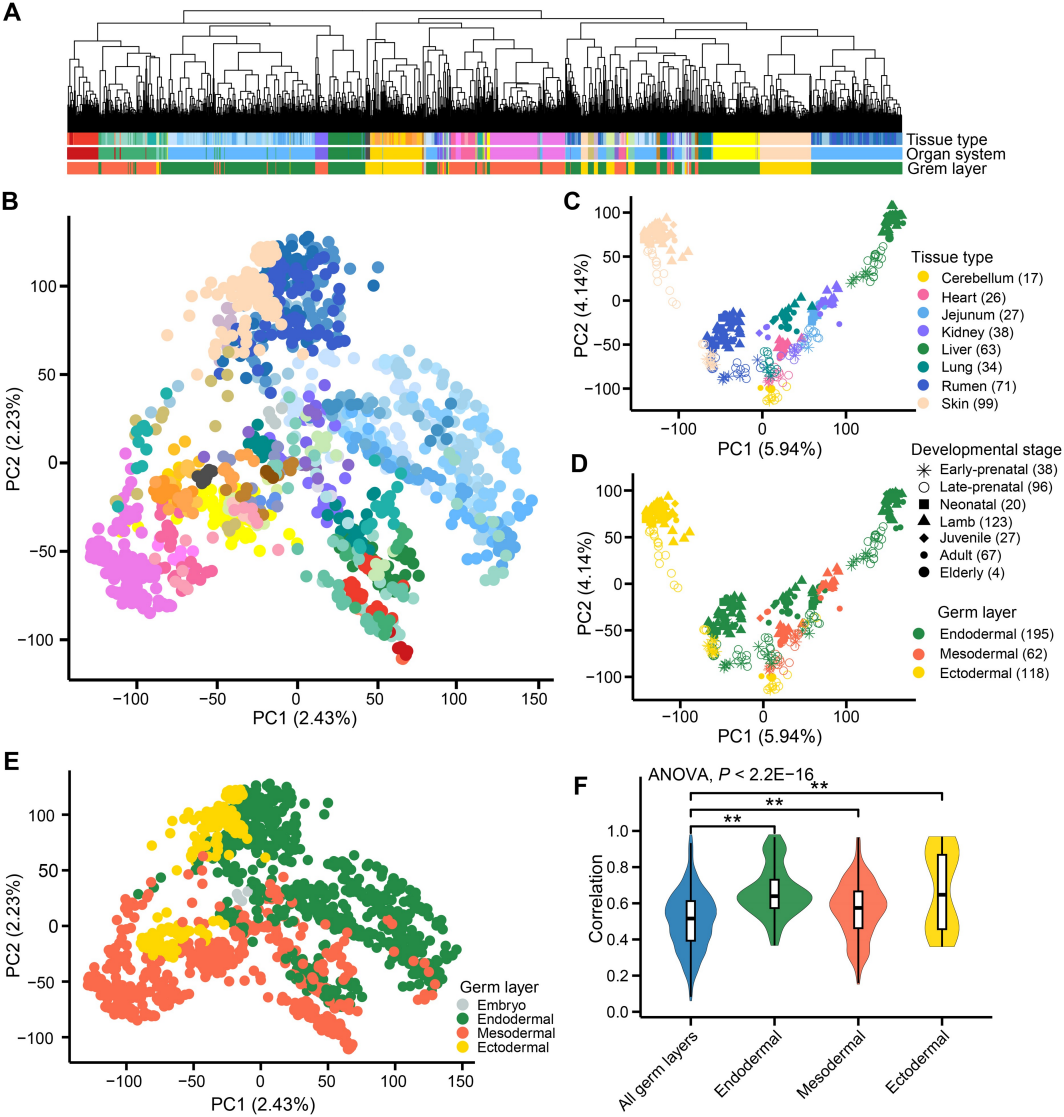
Clustering analysis of RNA-seq samples reveals tissue- and development-specific gene expression patterns **A**. Hierarchical clustering of 1413 RNA-seq samples based on distances (1−*r*). *r* is the Pearson correlation coefficient calculated from gene expression values (TPM) of 6000 genes with the highest expression variance across samples (measured by the SD of TPM). **B**. PCA of 1413 RNA-seq samples using the scaled expression [*i.e.*, log_2_ (TPM + 0.25)] of the same 6000 genes mentioned above. **C**. and **D**. PCA of 375 samples of 8 tissues derived from 3 germ layers, spanning all the 7 developmental stages, *i.e.*, early-prenatal (≤ E70), late-prenatal (> E70), neonatal (D0–D8), lamb (D8–M6), juvenile (M6–Y1.5), adult (Y1.5–Y7), and elderly (> Y7). The samples in (C) and (D) were colored to represent tissue type and germ layer, respectively. The shapes represent developmental stages. **E**. PCA of 51 tissues based on the median gene expression of the same 6000 genes mentioned above, representing embryo, endodermal, mesodermal, and ectodermal lineages. **F**. Comparison of gene expression correlations between tissues from the same and all germ layers. SD, standard deviation; TPM, transcripts per million; PCA, principal component analysis; PC, principal component.

Hierarchical clustering and principal component analysis (PCA) based on both gene expression and alternative splicing showed a clear separation of RNA-seq samples by tissue type (**Figure 2**A and B, Figure S3). Within most individual tissue types, samples could be clustered according to developmental stages, particularly between prenatal and postnatal stages (Figure S4). When focusing on eight tissues with large sample sizes (*n* > 15), spanning all the developmental stages and three germ layers, we found that in general, expression profiles of prenatal tissues were more similar to each other compared to those of postnatal tissues (Figure 2C and D). For instance, at the prenatal rather than postnatal stages, skin samples clustered together with many tissues (Figure 2C and D). Tissues from the same germ layer exhibited higher similarity in gene expression profiles than those from different germ layers, supporting that the embryonic origin influences tissue-specific gene expression (Figure 2E and F, Figure S5). All these results also support the reliability of this sheep dGEA resource for subsequent exploration of tissue- and development-specific biology in sheep.

### Discovery of tissue-specific gene expression and alternative splicing patterns

**Figure 3:**
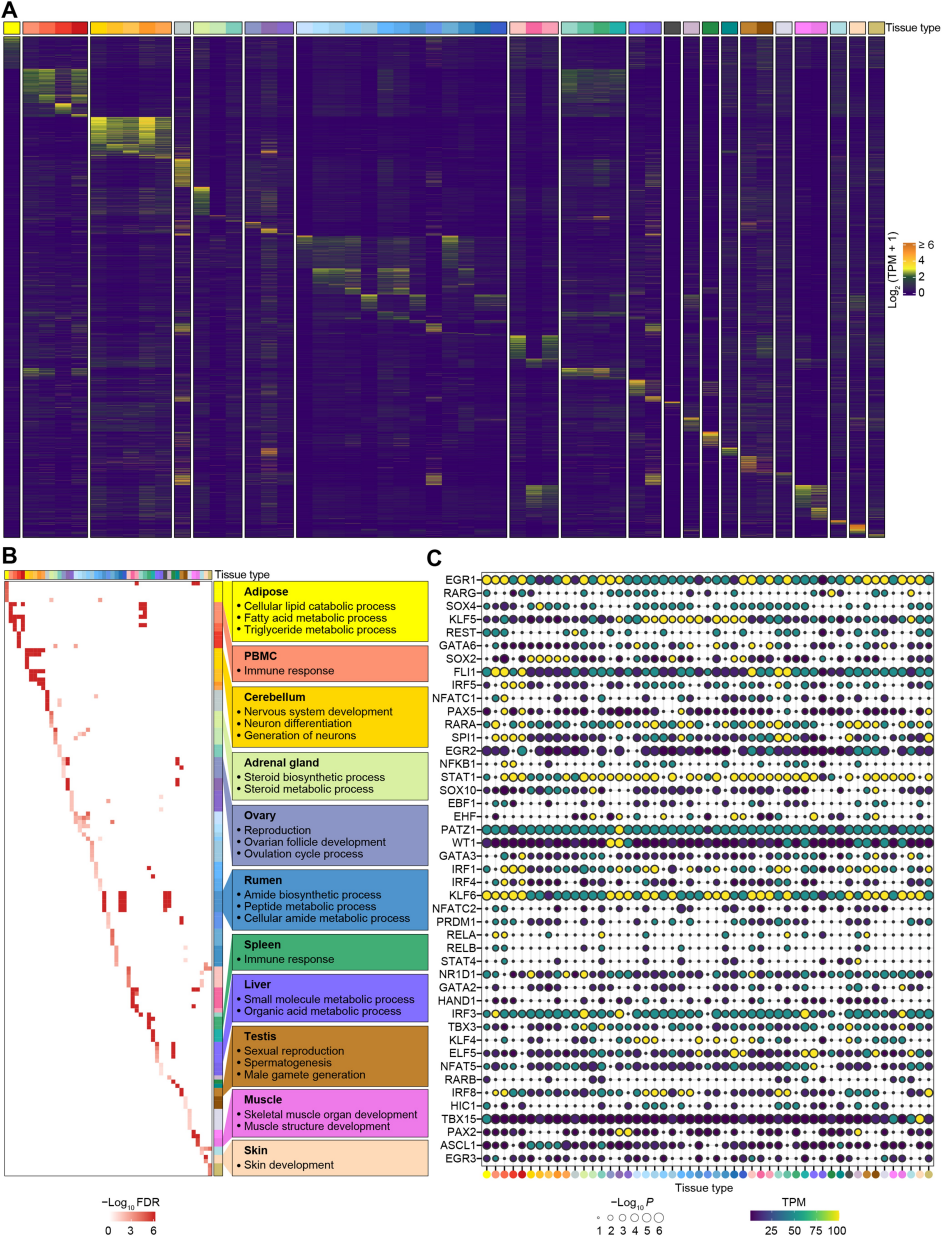
Tissue-specific gene expression **A**. Inter-individual median expression levels of tissue-specific genes across 51 tissues. Each row represents a gene, and each column represents an individual tissue. Color represents the median log_2_ (TPM + 1) of genes among all the samples in a tissue. **B**. GO terms significantly enriched (FDR < 0.05) in tissue-specific genes that were identified across 51 tissues, with examples of significant GO terms for several tissues presented in the left boxes. **C**. TFs with binding motifs significantly (*P* <  0.05) enriched in the promoters of tissue-specific genes. The x-axis represents the 51 tissues, with the same tissue color as presented in Figure 1A. GO, Gene Ontology; FDR, false discovery rate; TF, transcriptional factor.

By comparing a tissue with the remaining, we considered the top 5% of upregulated genes [based on false discovery rate (FDR) values] as tissue-specific genes in each of the 51 tissues (**Figure 3**A). Functional enrichment analysis of these tissue-specific genes revealed known biological and physiological functions of respective tissues (Figure 3B, Figure S6). For instance, adipose-specific genes were significantly (FDR < 0.05) enriched in cellular lipid catabolic process and fatty acid metabolic process, while cerebellum-specific genes were enriched in nervous system development and neuron differentiation (Figure 3B). We also detected genes with tissue-specific alternative splicing patterns (Figure S7A), whose functions also reflected known tissue biology (Figure S7B). For instance, 60 genes with specific alternative splicing in the cerebellum were significantly enriched in nervous system development. We further performed a motif enrichment analysis for promoters of these tissue-specific genes to detect the key transcription factors (TFs) determining tissue identity and functions. Enriched motifs and their target TFs revealed tissue-specific biology (Figure 3C). For example, GATA6 showed a specific expression in cardiac muscle and ventricle, and it has been proposed to play an important role in heart development [31]. Similar findings were observed for ARNT2 in the hypothalamus and FOXF1 in the lung, which were also supported by previous evidence [32] (Figure 3C). Some novel findings included PROX1 in the liver, TBX3 in the placenta, and PATZ1 in the oviduct.

### Developmental stage-specific genes in multiple tissues

**Figure 4:**
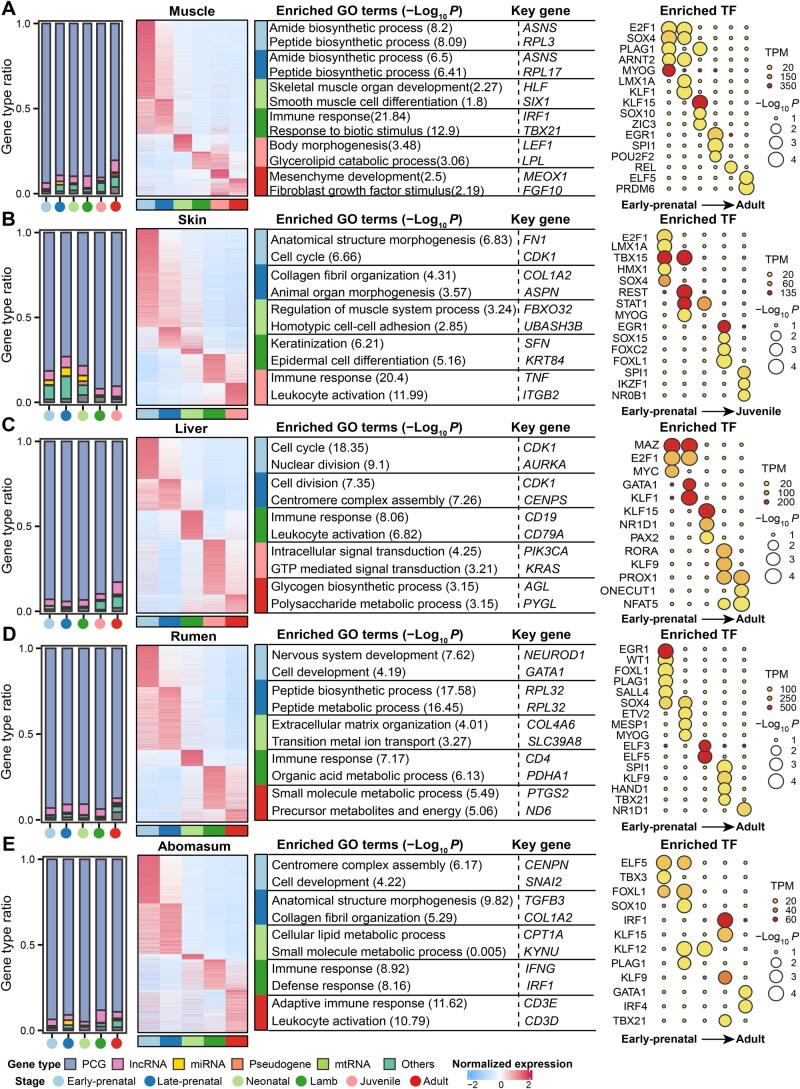
Features of developmental stage-specific genes in major tissues **A**. The panels for muscle from left to right illustrate the distribution of different gene types (PCG, lncRNA, miRNA, mtRNA, pseudogene, and others) among stage-specific genes, the expression level [log_2_ (TPM + 1)] of stage-specific genes across developmental stages, the GO biological processes enriched with stage-specific genes, and TFs with binding motifs significantly enriched in the promoters of stage-specific PCGs, respectively. **B**.−**E**. Similar to (A) but for skin (B), liver (C), rumen (D), and abomasum (E), respectively. PCG, protein-coding gene; lncRNA, long non-coding RNA; miRNA, microRNA; mtRNA, mitochondrial RNA.

We identified an average of 3150 developmental stage-specific genes across 20 tissues (over three biological replicates per developmental stage per tissue) (Figure S8). Prenatal tissues generally had a larger number of stage-specific genes than postnatal ones (Figure S8). Notably, in all tested tissues, PCGs were the predominant gene type among the stage-specific genes, followed by lncRNAs (**Figure 4**, Figures S9–S11). Developmental stage-specific genes were significantly enriched in biological processes related to the developmental, anatomical, and physiological features of respective tissues (Figure 4, Figures S9–S11). For instance, stage-specific genes in the muscle at early-prenatal, late-prenatal, neonatal, lamb, juvenile, and adult stages were significantly enriched in amide and peptide biosynthetic processes, muscle development and differentiation, immune processes, body morphogenesis, mesenchymal development and the regulation of fibroblast growth, respectively (Figure 4A). Additionally, in each of tissues, we identified key genes and TFs that play pivotal roles in these developmental transitions (Figure 4, Figures S9–S11). For instance, E2F1, SOX4, PLAG1, ARNT2, and MYOG are crucial for the proliferation and differentiation of muscle cells during the embryonic development [33–36]. In the neonatal stage, KLF15, SOX10, and ZIC3 serve as essential TFs for muscle development and the maintenance of the myogenic lineage [37–39]. EGR1, SPI1, and POU2F2 are significantly enriched at the lamb stage, responding to growth factors and immune signals [40,41]. In juveniles, REL is associated with immune responses and also plays a critical role in muscle development and regeneration [42]. In adults, ELF5 and PRDM6 are vital for regulating muscle cell differentiation (Figure 4A) [43].

### Gene clusters with distinct patterns of expression and TF regulation across developmental stages

**Figure 5:**
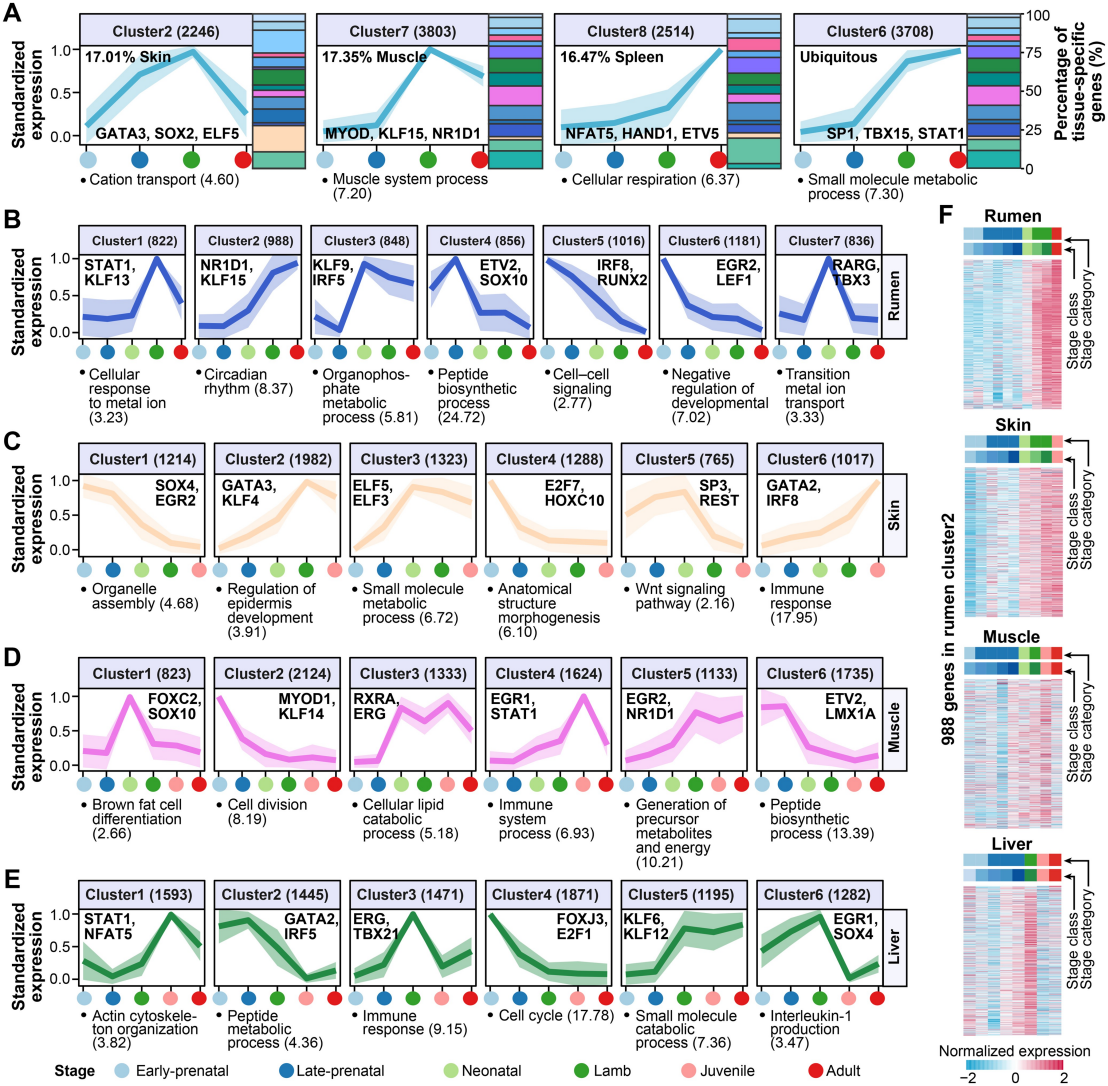
Time-course clustering analysis **A**. Trajectory clustering of all tissue-specific genes in all 20 tissues across four developmental stages, *i.e.*, early-prenatal, late-prenatal, lamb, and adult stages. These trajectories were grouped into 8 distinct gene clusters. Four representative clusters are displayed here, including Cluster2 (17.01% skin-specific genes), Cluster7 (17.35% muscle-specific genes), Cluster8 (16.47% spleen-specific genes), and Cluster6 (genes not dominated by any tissues) (see Figure S12 for other clusters). The TFs with binding motifs top significantly (FDR < 0.05) enriched in the promoters of genes in each cluster are listed at the bottom of the respective panel. The left y-axis represents the standardized expression values of tissue-specific genes in each cluster, while the right y-axis indicates the percentage of tissue-specific genes in each cluster. The developmental stages are displayed along the x-axis, with their colors shown at the bottom of the entire figure. The polygons represent mean ± SD. Numbers in the bracket at the top of each panel indicate the number of genes in the respective cluster. The top significantly (FDR < 0.05) enriched GO term for each cluster is listed at the bottom of the respective panel. **B**.−**E**. Clustering of developmental stage-specific genes in the rumen (B), skin (C), muscle (D), and liver (E), respectively. **F**. Heatmaps showing the normalized gene expression patterns of 998 genes detected in rumen Cluster2 in the rumen, skin, muscle, and liver, respectively. Stage class and stage category are consistent with Figure 1B.

To explore whether some genes share similar developmental expression patterns among the 20 tissues, we clustered genes according to their expression similarity in all 20 tissues across four developmental stages (*i.e.*, early-prenatal, late-prenatal, lamb, and adult stages) using a soft-clustering approach (c-means), resulting in 8 gene clusters (**Figure 5**A, Figure S12). For instance, 2246 genes in Cluster2 exhibited a peak of expression at the lamb stage, and then showed a quick decrease at the later stages. Among these, 17.01% were specifically and highly expressed in the skin and significantly enriched for cation transport and motifs of GATA3. GATA3 plays an important role in endothelial cell biology [44]. A total of 3803 genes in Cluster7 were upregulated at the late-prenatal stage and then downregulated at the lamb stage, and 17.35% of them had a specifically high expression in the muscle and were significantly enriched for the muscle system process and motifs of MYOD. MYOD participates in muscle cell differentiation [45]. The expression of 2514 genes in Cluster8 increased continuously from the early-prenatal stage, and 16.47% of them were specifically and highly expressed in the spleen and significantly enriched for cellular respiration and motifs of NFAT5. NFAT5 is engaged in inducible gene transcription during the immune response [46]. The expression of 3708 genes in Cluster6 gradually increased as development progressed and was upregulated sharply after the late-prenatal stage. Genes in this cluster were not dominated by tissue-specific genes of any tissues, which were significantly enriched with fundamental biological processes such as the small molecule metabolic process and motifs of SP1. SP1 is engaged in many cellular processes, including cell differentiation, cell growth, apoptosis, immune responses, and chromatin remodeling [47]. We also clustered genes using the same soft-clustering approaches within each of the 20 tissues (Figure 5B–E, Figures S13–S15). We took rumen, skin, muscle, and liver as examples in Figure 5B–E. We identified seven, six, six, and six gene clusters with distinct developmental expression patterns and enriched motifs in the rumen, skin, muscle, and liver, respectively (Figure 5B–E). For instance, the gene Cluster2 in the rumen encompassed 988 genes that displayed a gradual increase in expression as development progressed and were significantly enriched for circadian rhythm and motifs of NR1D1 (Figure 5B). Of note, the expression pattern of these genes shared certain similarities between the rumen and skin but not muscle or liver across developmental stages, consistent with the notation that both rumen and skin are epithelial tissues (Figure 5F). Beyond its well-known role in circadian rhythm regulation [48], *NR1D1* was also reported to be involved in metabolism [49], immune response [50], and cell differentiation [51]. We here proposed that NR1D1 might also serve as a developmental stage-specific TF across multiple tissues (Figure S16). However, functional follow-ups are required to confirm the role of *NR1D1* in developmental transitions.

### Gene co-expression analysis enhances functional annotation of the sheep genes

**Figure 6:**
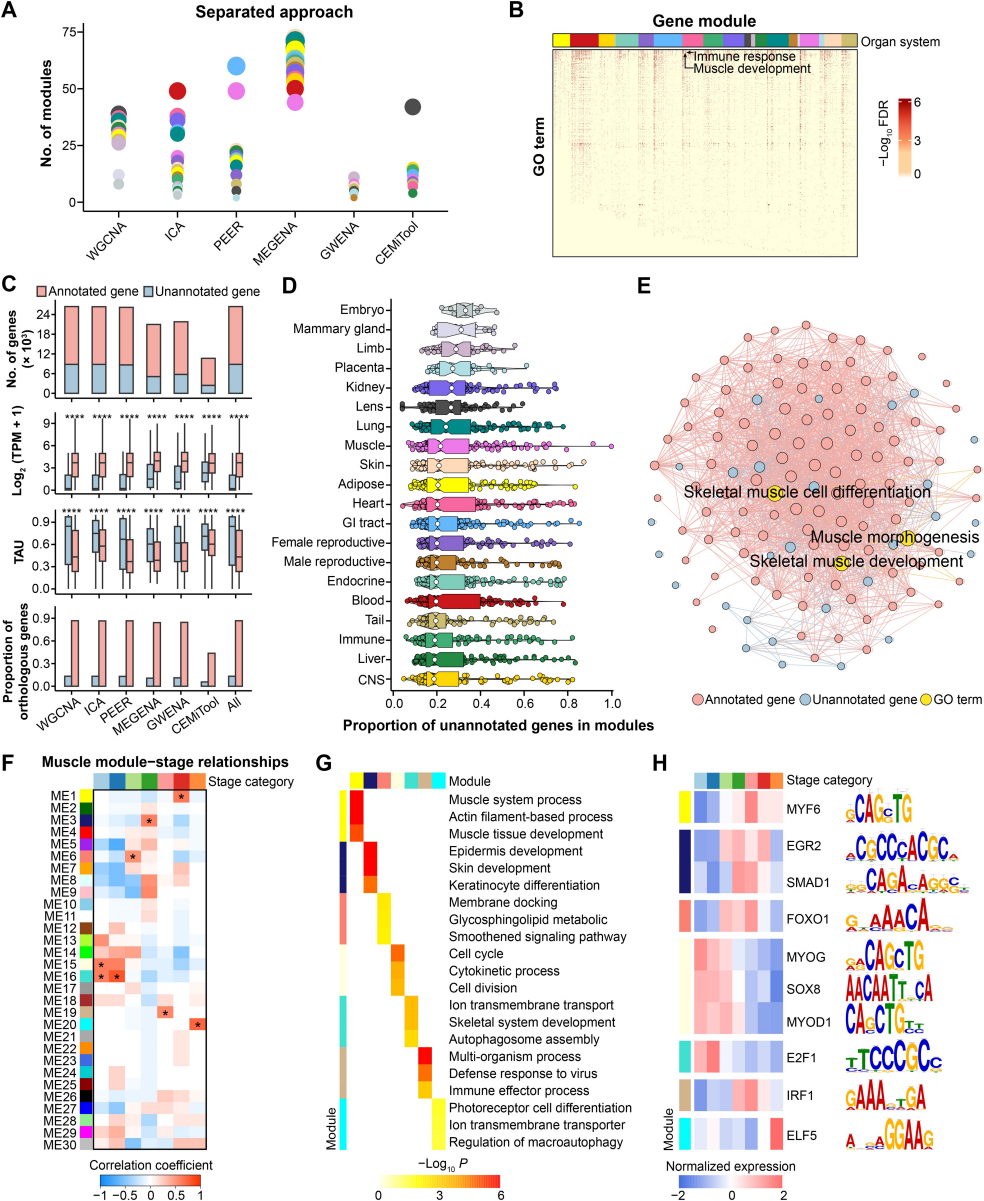
Gene co-expression network analysis across tissues and developmental stages **A**. Number of gene modules detected in each of 20 tissues by 6 complementary gene co-expression approaches, including WGCNA, ICA, PEER, MEGENA, GWENA, and CEMiTool. Each dot represents a tissue, and the dot size represents the number of detected gene modules. **B**. Significantly enriched GO terms (FDR < 0.05) in gene modules across 20 tissues. **C**. Comparison of genes with and without GO annotation (referred to as “annotated genes” and “unannotated genes”, respectively) in gene co-expression modules across different features. The gene functional annotation was based on the GO database (release January 18, 2022). “All” means the combined results from the six co-expression methods. The plots from top to bottom are number of genes, expression level, TAU values, and the proportion of orthologous genes in humans. Significant differences between annotated and unannotated genes are obtained by a two-sided *t*-test. ****, *P* < 0.0001. **D**. The proportion of unannotated genes in each of the gene co-expression modules across 20 tissues. **E**. An example of gene co-expression modules in muscle detected by WGCNA includes 34 unannotated and 106 annotated genes. All these genes are significantly (FDR < 0.05) enriched in muscle morphogenesis, skeletal muscle cell differentiation, and skeletal muscle development, which are denoted as yellow. The edges between genes represent Pearson correlation coefficients (*r* ≥ 0.7, *P* < 0.05) of expression levels across all the 129 samples in muscle. The circle size corresponds to the interaction degree. **F**. Correlations between gene modules and developmental stages in muscle. The statistical significance of the muscle module–developmental stage relationship is corrected for multiple testing using the FDR method. The stars denote FDR < 0.05 (muscle-specific modules). **G**. The top significantly enriched GO terms (*P* < 0.05) of developmental stage-specific modules in muscle. **H**. The top enriched binding motifs and expression patterns of TFs of developmental stage-specific modules in muscle. WGCNA, weighted gene co-expression network analysis; ICA, independent component analysis; PEER, probabilistic estimation of expression residuals; MEGENA, multiscale embedded gene co-expression network analysis; GWENA, gene whole co-expression network analysis; CEMiTool, co-expression modules identification tool.

To improve the functional annotation of sheep genes using such a large and diverse transcriptome dataset, we performed gene co-expression analyses using six complementary approaches in two scenarios: (1) all the tissues (integrated approach) and (2) single tissue (separated approach) [52–57]. In total, we identified 237 and 2437 gene modules from the integrated and separated approaches, respectively (**Figure 6**A, Figure S17A–C). To evaluate the similarity and difference between gene modules identified by different methods, we calculated the Jaccard similarity coefficient (referred to here as the module sharing index), which is the ratio of the intersection to the union of genes between module pairs (Figure S17D). We specifically compared modules identified by weighted gene co-expression network analysis (WGCNA) with those identified by the remaining methods (Figure S17E and F). In general, modules detected by these methods exhibited a low module sharing index, reflecting that they are complementary approaches for gene co-expression analyses, as demonstrated by recent benchmarks that there is no single best co-expression analysis method [58,59]. Among all the 2437 modules detected by the single-tissue approach, 49.45% (1205) were significantly enriched for at least one Gene Ontology (GO) term (FDR < 0.05) (Figure 6B). Cross-tissue module preservation analysis identified 26 modules which were strongly preserved across tissues (Zsummary > 10), while 9 were tissue-specific with a weak preservation (Zsummary < 2; Figure S18). Tissue-shared modules are more likely to be enriched in fundamental biological processes (*e.g.*, cell cycle, chromatin assembly, and translation), while tissue-specific modules tend to be enriched in tissue-relevant biology. For instance, two muscle-specific modules were significantly enriched in muscle development (*P* = 7.98 × 10^−6^) and immune response (*P* = 2.22 × 10^−43^) (Figure 6B).

Among all 26,423 genes in the 2437 modules, 8813 (33.35%) genes had no functional annotation in the current GO database (release January 18, 2022), which were hereafter referred to as “unannotated genes”. Compared to annotated genes, unannotated genes had lower expression and higher tissue specificity, and were less likely to have human orthologous genes (Figure 6C). The proportion of unannotated genes in co-expression modules was distinct across tissues (Figure 6D). The embryo and mammary gland had the highest proportions of unannotated genes (an average of 33.15% and 31.14%, respectively). In contrast, central nervous system (CNS) and liver had the lowest proportions (an average of 18.70% and 19.01%, respectively). Of note, a total of 2861 unannotated PCGs were able to be assigned to at least one co-expression module, contributing to their functional annotation (Figure S17G and H). For instance, in a module detected in muscle by WGCNA, 34 unannotated genes were co-expressed with 106 genes that were functionally annotated with muscle morphogenesis, skeletal muscle cell differentiation, and skeletal muscle development by the GO database (Figure 6E). We could thus infer that these 34 unannotated genes are more likely to have functions on muscle development in sheep.

Within each of the 20 tissues, we further linked the WGCNA-detected co-expression modules to developmental stages. We took muscle as an example in Figure 6F–H. Among 30 co-expression modules, 7 were detected as developmental stage-specific modules (Figure 6F). For instance, genes in module-1 (ME1) were significantly (FDR < 0.05) upregulated at the adult stage and enriched in the muscle system process, actin filament-based process, and muscle tissue development (Figure 6G). The following motif enrichment analysis of genes in developmental stage-specific modules revealed biologically relevant TFs, which also showed developmental stage-specific expression (Figure 6H). For instance, MYF6, acting as a myogenic factor that prompts fibroblasts to differentiate into myoblasts [60], was observed with specific expression in adults. MYOG and MYOD1, well-recognized TFs essential for the development of functional skeletal muscle [61,62], were highly expressed at the early-prenatal stage. Additionally, FOXO1, a key player in myogenic growth and differentiation [63,64], demonstrated developmental stage-specific expression in neonates. Furthermore, the analysis proposed several novel TFs for muscle development for future functional validations. For instance, SOX8, involved in regulating embryonic development and determining cell fate, exhibited developmental stage-specific expression in muscle at the early-prenatal stage. In contrast, SMAD1, which plays a role in developmental processes and immune responses, showed developmental stage-specific expression in muscle at the lamb stage. Additionally, IRF1, functioning as an activator for genes implicated in innate and acquired immune responses, showed developmental stage-specific expression in muscle at the juvenile stage. Together, these findings advanced our understanding of the development-dependent roles of TFs in muscle development and function in sheep. In addition, we found that the STAT [65] and KLF [66] families played essential roles in regulating the development and function of the liver and kidney, respectively (Figure S19).

### Leveraging dGEA to interpret rumen evolution and monogenic traits in sheep

**Figure 7:**
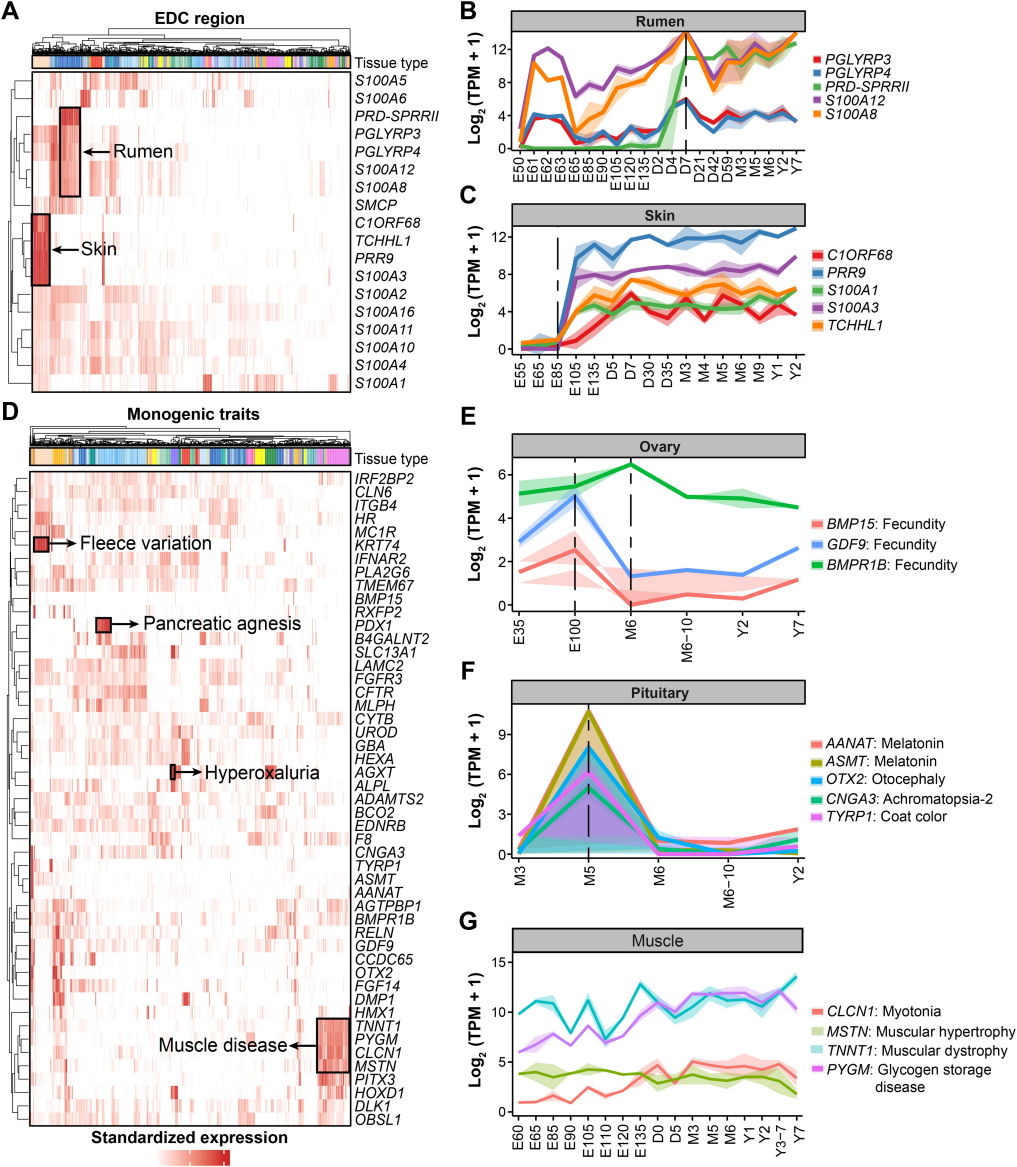
Leveraging dGEA to interpret ruminant-relevant genes and monogenic traits in sheep **A**. Expression levels of 18 genes in the EDC region across 51 tissues. The gene expression levels are normalized as TPM. The y-axis represents the 18 EDC genes, and the x-axis represents the 51 tissues. **B**. The developmental expression patterns of five EDC genes with specific expression in rumen. **C**. The developmental expression patterns of 5 EDC genes with specific expression in skin. **D**. Expression levels of 49 genes associated with sheep monogenic traits across the 51 tissues. **E**.−**G**. Developmental expression patterns of genes with specific expression in ovary (E), pituitary (F), and muscle (G), respectively. The monogenic traits associated with each gene are listed next to it. EDC, epidermal differentiation complex.

Previous studies have identified a group of genes known as the epidermal differentiation complex (EDC), which are responsible for epithelial tissue (*e.g.*, rumen, skin, and wool) development and repair by regulating the terminal differentiation program of the keratinocytes through a series of coordinated and inter-dependent signal transduction pathways [1,67–69]. We examined expression patterns of all 18 EDC genes across tissues and developmental stages. Among these, *PGLYRP3*, *PGLYRP4*, *PRD-SPRRII*, *S100A12*, and *S100A8* were specifically and highly expressed in the rumen, while *C1ORF68*, *PRR9*, *S100A1*, *S100A3*, and *TCHHL1* were specifically and highly expressed in the skin (**Figure 7**A). All five rumen-specific EDC genes showed similar developmental stage-specific expression patterns as well as highest expression levels at D7 (Figure 7B). All the five skin-specific genes have little or no expression at the early-prenatal stages (*i.e.*, embryos at E55–E85) and then rapidly upregulated after E85, partially because the number of primary follicles increases and the secondary follicles start to form (Figure 7C).

We examined the Online Mendelian Inheritance in Animals (OMIA) [70] to explore expression patterns of 49 causal genes of 48 monogenic traits across tissues and developmental stages in sheep. Many of them showed strong tissue- and developmental stage-specific expression patterns (Figure 7D, Figure S20). For instance, *BMPR1B*, *BMP15*, and *GDF9* are proposed as causal genes of fecundity in sheep, which were specifically and highly expressed in the ovary (Figure 7D). However, the developmental expression patterns of *BMP15* and *GDF9* were different from that of *BMPR1B*. *BMP15* and *GDF9* showed the highest expression at E100 and the lowest expression at M6, whereas *BMPR1B* showed the highest expression level at M6 (Figure 7E). Of note, our analysis also proposed novel trait–tissue associations for future functional validations. For example, causal genes of melatonin (*AANAT*), melatonin (*ASMT*), achromatopsia-2 (*CNGA3*), otocephaly (*OTX2*), and coat color (*TYRP1*) were specifically and highly expressed in the pituitary. These genes showed similar developmental patterns, with little expression at M3 and a quick peak at M5 but then downregulated sharply afterward, indicating their specific roles in the pituitary at M5 (Figure 7F). Similarly, five causal genes of muscle-relevant diseases, including *MSTN* and *DLK1* of muscular hypertrophy, *TNNT1* of muscular dystrophy, *CLCN1* of myotonia, and P*YGM* of glycogen storage disease, exhibited a strong muscle-specific expression, but showed different expression patterns as development progressed (Figure 7G). Furthermore, dGEA can also help interpret the tissue and developmental expression patterns of genes already annotated by GO terms in sheep. For instance, 12 genes engaged in the lipid metabolic process were explicitly and highly expressed in adipose (Figure S21A). The expression of these genes gradually increased from E85 and reached a peak at E132 before decreasing sharply, then rebounding at M5, and stabilizing at a high expression level from that point onward (Figure S21B).

### Genetic variants of complex traits were enriched in tissue- and developmental stage-specific genes

**Figure 8:**
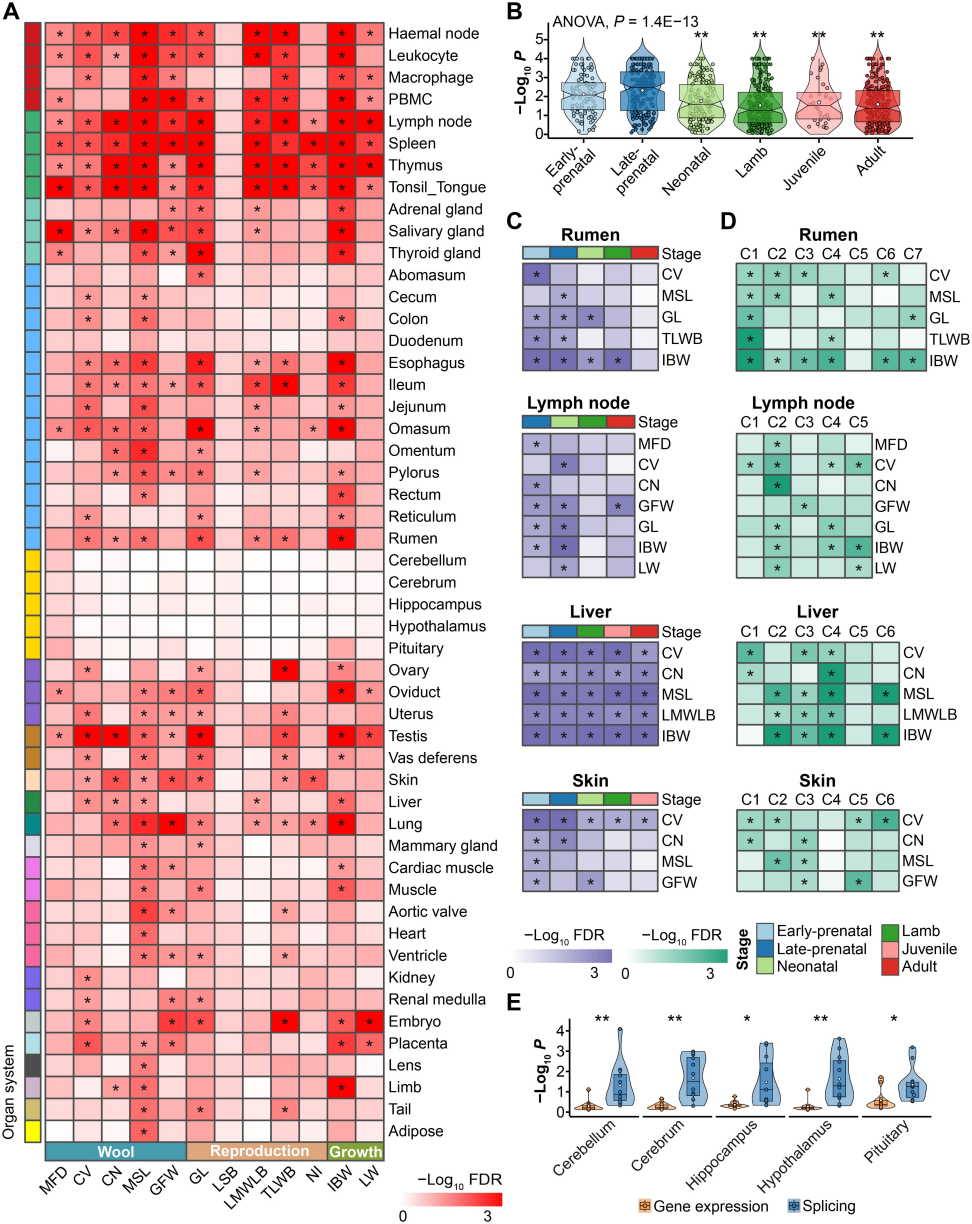
Enrichment of GWAS signals in genes with specific expression and splicing patterns across various tissues and developmental stages **A**. GWAS signal enrichment results of 12 complex traits in 51 tissues. The color corresponds to the enrichment degree (*i.e.*, −log_10_ FDR), which was computed by a sum-based GWAS signal enrichment analysis based on the tissue-specific genes (*, FDR < 0.05). The x-axis represents the 12 complex traits (MFD, CV, CN, MSL, GFW, GL, LSB, LMWLB, TLWB, NI, IBW, and LW). **B**. Comparison of GWAS signal enrichment results (−log_10_  *P* value) of all 12 complex traits in genes with developmental stage-specific expression patterns. The ANOVA test was used to determine whether GWAS enrichment was significantly different across developmental stages, and the late-prenatal stage was selected as the target stage for pairwise comparisons with other stages (**, *P* < 0.01). **C**. Similar to (A), but for developmental stage-specific genes in rumen, lymph node, liver, and skin, respectively. **D**. Similar to (C), but for gene clusters associated with developmental stages. **E**. Comparison of GWAS signal enrichment results (−log_10_  *P* value) between genes with tissue-specific expression and alternative splicing across cerebellum, cerebrum, hippocampus, hypothalamus, and pituitary. The paired *t*-test was used to determine whether GWAS signal enrichment was significantly different between these two groups of genes in each tissue (*, *P* < 0.05; **, *P* < 0.01). GWAS, genome-wide association study; MFD, mean fiber diameter; CV, coefficient of variation of the fiber diameter; CN, crimp number; MSL, mean staple length; GFW, greasy fleece weight; GL, gestation length; LSB, litter size at birth; LMWLB, litter mean weight per lamb born; TLWB, total litter weight at birth; NI, number of mating pregnancy; IBW, individual birthweight; LW, live weight.

To explore whether dGEA can improve our understanding of the genetic and developmental architecture underlying complex traits in sheep, we first conducted single-marker GWAS for 12 complex traits of economic importance in a Merino sheep population (*n* = 1639), including 5 wool traits, 5 reproductive traits, and 2 growth traits. We then tested the enrichment of GWAS signals in tissue- and developmental stage-specific genes and gene clusters detected above (**Figure 8**; Table S2). As shown in Figure 8A, GWAS signals of complex traits were significantly (FDR < 0.05) enriched in genes with tissue-specific expression. In general, immune/blood-specific genes showed the highest enrichment for all traits, followed by gastrointestinal (GI) tract-specific genes. In contrast, CNS-specific genes showed no significant enrichment for any traits (Figure 8A). The muscle-specific genes showed significant enrichment for GWAS signals of mean staple length (MSL), gestation length (GL), and individual birthweight (IBW). Compared to other traits, GWAS signals of wool traits such as coefficient of variation of the fiber diameter (CV), crimp number (CN), MSL, and greasy fleece weight (GFW) were more likely to be enriched in skin-specific genes. Of note, GWAS signals of all the complex traits showed higher enrichment in genes with specific expression in prenatal tissues than those in postnatal tissues (Figure 8B). For instance, genes with specific expression in the prenatal rather than adult rumen, lymph node, and skin were significantly associated with many traits (Figure 8C). Within the rumen, genes in Cluster1, the expression of which showed a peak at the lamb stage and quickly decreased from then on (Figure 8B), were significantly enriched for five traits, including CV, MSL, GL, total litter weight at birth (TLWB), and IBW (Figure 8D), while all the gene clusters, except for Cluster5, were significantly associated with IBW, indicative of the complexity of body growth regulation (Figure 8D).

Furthermore, we conducted GWAS signal enrichment analyses using alternative splicing results (Figure S22; Table S3). Like gene expression, genes with specific splicing patterns in many tissues were significantly enriched for GWAS signals of mean fiber diameter (MFD), MSL, TLWB, and IBW (Figure S22A). Of note, although genes with specific expression in CNS (cerebellum, cerebrum, hippocampus, hypothalamus, and pituitary) showed no enrichment for GWAS signals of any complex traits studied (Figure 8A), genes with specific splicing patterns in CNS were significantly enriched for GWAS signals of MFD, CV, TLWB, and IBW (Figure 8E). This finding proves the importance of studying alternative splicing of genes in CNS for understanding molecular mechanisms behind complex traits [71–73].

### Integrating GWAS fine-mapping results and dGEA reveals key genes for complex traits in sheep

**Figure 9:**
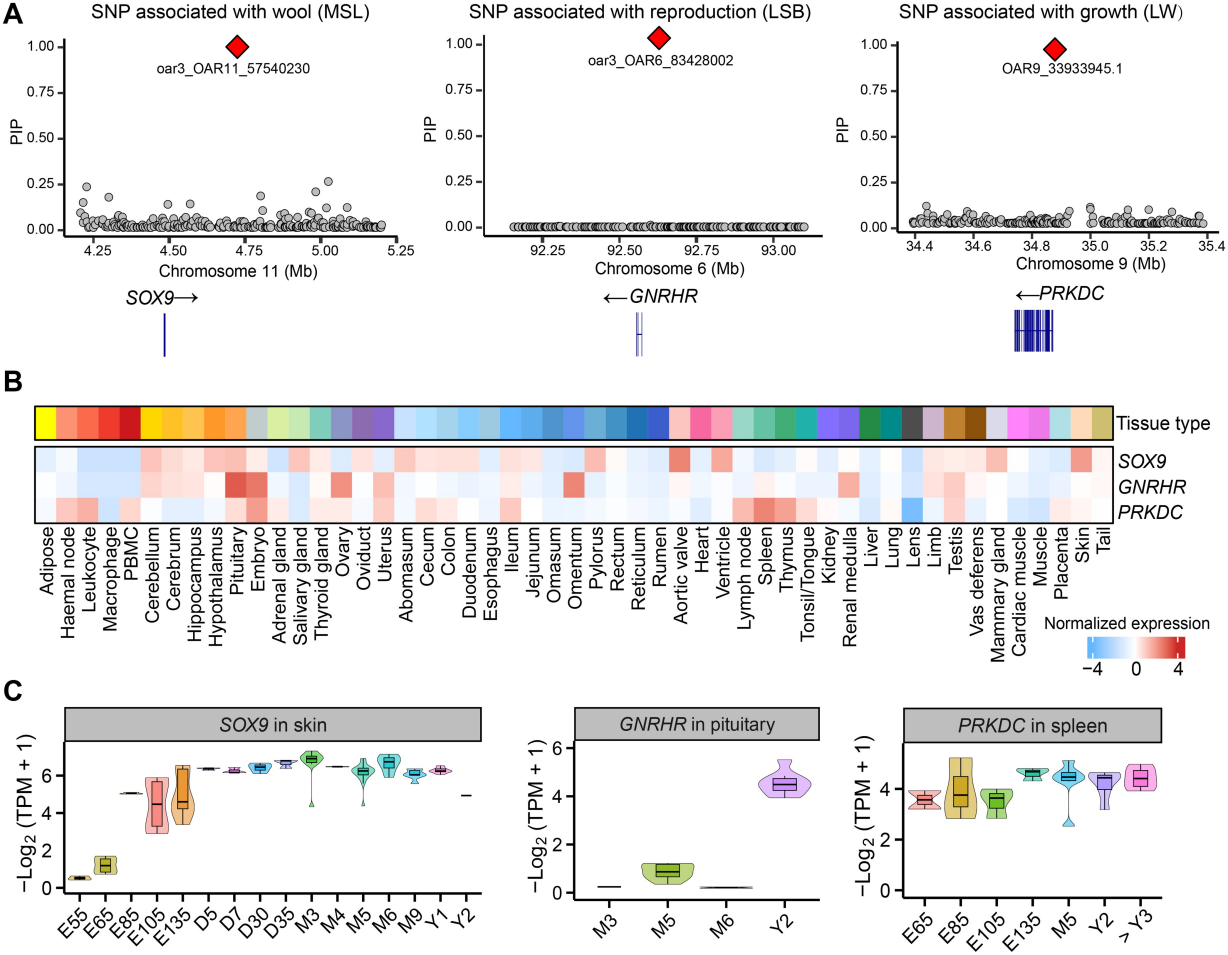
Integrative analysis of fine-mapping and sheep dGEA reveals key genes associated with complex traits in sheep **A**. Fine-mapping for significant SNPs associated with wool (MSL, left), reproduction (LSB, middle), and growth (LW, right) traits in sheep. **B**. Tissue-specific expression patterns of key genes. **C**. Stage-specific expression patterns of key genes. SNP, single-nucleotide polymorphism; PIP, posterior inclusion probability.

We conducted fine-mapping analysis on QTL regions, *i.e.*, within ± 500 kb of lead single-nucleotide polymorphisms (SNPs), to identify potential causal variants for sheep wool, growth, and reproductive traits (Tables S4 and S5). In total, 196 SNPs were detected as causal variants [posterior inclusion probability (PIP) > 0.9, Table S6]. By comprehensively integrating these fine-mapping results with dGEA, we proposed several promising candidate genes for complex traits in sheep (**Figure 9**). For example, *SOX9* was associated with MSL and specifically expressed in skin. Its expression level increased gradually during the process of skin development. *GNRHR* showed a significant association with litter size at birth (LSB) and specifically and highly expressed in the pituitary of adult sheep. *PRKDC* was associated with live weight (LW) and showed the highest expression level at E135 in the spleen compared to other stages.

## Discussion

In this study, we constructed a high-resolution multi-tissue developmental gene expression atlas in sheep by integrating high-quality RNA-seq data from 1413 samples, covering 51 tissues spanning 14 developmental time points from early organogenesis to adulthood, representing the largest study to date on multi-tissue developmental gene expression in sheep. Compared to the previous sheep gene expression atlas reported by Emily et al. in 2017 [21], we produced and analyzed approximately three times more RNA-seq data, covering nine additional developmental stages. We systematically explored the dynamic landscape of gene expression across the process of multi-tissue development and proposed key genes and gene regulatory networks that are essential for development transitions. Additionally, by integrating dGEA with GWAS results, we provided novel insights into the genetic and developmental basis of complex traits in sheep. The dGEA will serve as an invaluable resource for genetic and genomic research in sheep.

Integrating GWAS fine-mapping results and dGEA revealed several candidate genes for complex traits in sheep, including *SOX9* for MSL, *GNRHR* for LSB, and *PRKDC* for LW. *SOX9* has been reported to be essential for outer root sheath differentiation and the formation of the hair stem cell compartment [74]. Previous studies have demonstrated that the proper expression of gonadotropin-releasing hormone receptors (GnRHRs) by pituitary gonadotropes is critical for maintaining maximum reproductive capacity. In addition, GnRHR could influence the litter size of pigs [75] and goats [76]. Therefore, we proposed GnRHR as a candidate gene for litter size in sheep. A previous GWAS by Horodyska et al. [77] revealed that *PRKDC* was associated with feed conversion efficiency and growth rate in pigs. It was also reported to be associated with body conformation traits in sheep [78]. We thus proposed that *PRKDC* could be a promising candidate gene for LW in sheep. In our study, we found that gene expression patterns were generally conserved across breeds regarding tissue types and developmental stages, indicating that the phenotypic variation introduced by breed-specific gene expression is limited compared to tissue- and developmental stage-specific. However, in future study, it still is of interest to explore breed-specific gene expression and regulation by collecting large-scale samples at matching biological contexts (*e.g.*, tissue, cell, and development) and environmental conditions (*e.g.*, diet and pathogen exposure) across distinct breeds.

Although the current dGEA serves as a valuable resource for sheep genetics and genomics studies, we notice some limitations and give relevant perspectives here. Tissues and embryonic samples before E40 were investigated in this study. Despite challenges in distinguishing anatomical structures and fragile nature of samples at the early developmental stage, collection of samples before E40 will be crucial for understanding early developmental processes, such as cell differentiation and the establishment of foundational organ systems [21]. Our initial assumption was that genomic variations influence complex traits by modifying the expression of genes in specific tissues and cell types or at specific developmental stages. As reported by the human GTEx [13] and FarmGTEx Consortia [19,20], the majority of expression quantitative trait loci (eQTLs) are *cis*-variants, physically close to transcript start sites (TSSs) of genes. We thus concentrated on investigating *cis*-variants rather than *trans*-variants of tissue/development-specific genes by including variants 20 kb upstream and downstream of respective genes. To explore *trans*-variants, a large number of samples (*n* > 1000) are often required due to their relatively minor effects [8]. As tissues are a mixture of different cell types, the cell type composition of tissues being analyzed could confound our interpretation of some results. As we showed in Figure 8A, leukocytes, macrophages, and peripheral blood mononuclear cells (PBMCs) had distinct enrichment results across wool, reproduction, and growth traits. Therefore, it will be crucial to generate pure bulk and/or single-cell expression data to investigate cell type-specific gene expression and regulation across developmental stages. In the future, integrating more advanced technologies, such as single-cell omics [79–81], spatial transcriptomics [82,83], long-read RNA-seq [84], or epigenomics [85], will further refine the functional annotation of sheep genes. This will help us further understand mammalian development and determine the causal molecular factors that drive organ development and influence organismal phenotypes.

## Materials and methods

### Tissue sample collection

In this study, we totally collected 426 tissue samples from two populations, including Chinese Merino and Australian Merino. The newly generated RNA-seq dataset from the Chinese Merino sheep consisted of 277 samples. The 12 embryos were collected from pregnant ewes at four embryonic time points (*i.e.*, E65, E85, E105, and E135; three biological replicates per time point). Specifically, we obtained 41 samples from E65 across 17 tissues, 78 samples from E85 across 29 tissues, 79 samples from E105 across 29 tissues, and 79 samples from E135 across 29 tissues.

The newly generated RNA-seq dataset from the Australian Merino sheep comprised 149 samples. Tissue samples were collected at eight different developmental time points, including four embryonic and four postnatal time points. The 65 embryonic samples included five E30 embryos for esophageal tube, five E50 embryos for rumen, five E90 embryos for four tissues (reticulum, rumen, omasum, and abomasum), and five E120 embryos for seven gut tissues (reticulum, rumen, omasum, abomasum, duodenum, cecum, and colon). The 84 postnatal samples consisted of the same seven gut tissues, were collected from three neonatal lambs (before their first feed), three lambs at W3 (before rumination, with no grass present in their GI tract), three at W6 (once rumination established), and three at W12 (after weaning). All tissues were collected immediately after the euthanasia of the ewes and stored in liquid nitrogen. The total RNA was extracted using the TRIzol reagent (Catalog No. 15596026CN, Thermo Fisher Scientific, Waltham, MA), and sequencing libraries were prepared with the NEBNext Ultra RNA Library Prep Kit (Catalog No. E7530, Illumina, San Diego, CA). The RNA libraries were sequenced to generate 150-bp paired-end (PE150) reads using the Illumina NovaSeq 6000.

Furthermore, we downloaded 1131 publicly RNA-seq samples (fastq files) within detailed metadata of developmental stages and tissue types from the National Center for Biotechnology Information (NCBI) Sequence Read Archive (SRA: PRJEB19199, PRJEB6169, PRJNA485657, PRJNA414087, PRJNA287258, PRJNA309284, PRJNA315011, PRJNA362606, PRJNA395156, PRJNA432669, PRJNA450309, PRJNA490799, PRJNA504353, PRJNA524671, PRJNA526287, PRJNA596252, PRJNA638028, PRJNA665351, PRJNA729910, PRJNA736945, PRJNA182914, PRJNA244152, PRJNA378408, PRJNA421633, PRJNA507468, PRJNA565839, PRJNA595784, PRJNA625152, PRJNA639853, and PRJNA705554), which are publicly accessible at https://www.ncbi.nlm.nih.gov/sra. In total, we analyzed 1557 RNA-seq samples in this study. Based on known biology [8], we classified 51 tissues and cell types into 20 tissue categories. The details of all the RNA-seq samples are summarized in Table S1.

### Quantification of gene expression and alternative splicing

We analyzed all the 1557 RNA-seq samples using the following bioinformatics pipeline. First, we removed adaptors and trimmed low-quality reads using Trimmomatic (v0.39) with the parameters of “TruSeq3-PE.fa:2:30:10 LEADING:3 TRAILING:3 SLIDINGWINDOW:4:15 MINLEN:36” [86]. We then aligned the clean reads to the sheep (Ovis aries) reference genome (Oar_rambouillet_v1.0, Ensembl v104) using STAR (v2.7.8a) [87] with the parameters of “--quantMode GeneCounts --chimSegmentMin 10 --chimOutType Junctions --chimOutJunctionFormat 1 --outSAMtype BAM SortedByCoordinate --outSAMunmapped Within --readFilesCommand zcat --outFilterMismatchNmax 3”, resulting in an averaged uniquely mapped rate of 81.79% (Table S1). For downstream analyses, we kept 1470 samples with a uniquely mapped rate ≥ 0.6 and clean read count > 10 million. We extracted raw read counts of 26,478 Ensembl (v104) genes by featureCounts (v2.0.1) [88] and obtained their normalized expression (*i.e.*, TPM) using Stringtie (v2.1.4) [89], Salmon (v1.3.0) [90], and Kallisto (v0.45.1) [91]. We removed six samples with less than 40% of all the genes expressed (TPM ≥ 0.1), and three tissues with sample size < 3, resulting in 1461 samples. We filtered genes that were not expressed in any samples, resulting in 26,423 genes for subsequent analyses. We performed the hierarchical clustering of all the RNA-seq samples using the *hclust* function in R package (v4.2.2). The distance between samples was measured by 1−*r*, where *r* was Pearson correlation coefficient based on the normalized quantile log_2_ (TPM + 0.25) of 6000 genes with the highest variance across samples. We also visualized these samples using the PCA approach implemented in the *prcomp* function in R package. After filtering out 48 outliers based on sample clustering, we eventually kept 1413 samples for subsequent analysis. For alternative splicing, we applied Leafcutter (v0.2.9) [92] to quantify intron excision levels using exon–exon junction reads from RNA-seq data in each of the tissue types as described previously [20]. We normalized intron excision levels as percent splicing (PSI) values for the downstream differential splicing (DS) analysis *via* the script “leafcutter_ds.R” in the LeafCutter package.

### Tissue-specific gene expression analysis

We employed tspex to quantify the tissue specificity of gene expression by calculating TAU (τ) values [93]. By comparing a tissue with the remaining, we conducted gene differential expression analysis using the R package edgeR (v3.40.2) [94], and considered the top 5% of upregulated genes in the target tissue based on FDR values as its tissue-specific genes [8]. We then performed the functional enrichment analysis of tissue-specific genes for biological process (BP) terms in the GO database using both R package clusterProfler [95] and gene set enrichment analysis (GSEA) [96], and considered FDR < 0.05 as significant. We conducted motif enrichment analysis on the promoter regions of tissue-specific genes using MEME software [97], defined as 1500 bp upstream to 500 bp downstream of TSS. The analysis was conducted based on the JASPAR (2024) core non-redundant vertebrate motifs [98] and the HOCOMOCO (v11) human motif set [99]. Motifs were considered significant when FDR < 0.05.

### Development-specific and time-course gene expression analysis

To identify genes with developmental stage-specific expression, we carried out differential expression analysis between one stage and the rest in each of the 20 tissues using the R package edgeR (v3.40.2) [94], where the sample size of each stage pretissue was > 3. We considered genes with log_2_ fold change > 2 and FDR < 0.05 as developmental stage-specific genes. Within each of the 20 tissues, we then clustered genes based on their expression levels across developmental stages using the soft-clustering approach (c-means) implemented in the R package mFuzz (v2.32.0) [100,101]. For this analysis, we only considered genes with development-specific expression detected above. The number of gene clusters was individually determined in each of 20 tissues using the minimum centroid distance measurement [100,101].

### Gene co-expression network analysis

We applied six complementary methods with default parameters, including WGCNA (v1.69) [52], GWENA (v1.8.0) [53], ICA (v1.0.2) [54], PEER (v1.3) [55], MEGENA (v1.3.7) [56], and CEMiTool (v1.8.3) [57], to identify modules of co-expressed genes based on the quantile normalized gene expression. Gene co-expression clustering methods, such as WGCNA, GWENA, and CEMiTool, aimed to identify non-overlapping co-expressed gene modules. In contrast, matrix factorization methods, such as ICA, PEER, and MEGENA, used factor loadings as module eigengene profiles, resulting in larger modules that included overlapping sets of genes. We further removed genes with zero standard deviation (SD) in each tissue to ensure the robustness of the analysis. Functional enrichment analysis of gene co-expression modules was conducted by using clusterProfiler (v4.0) [95], followed by visualization using Gephi (v0.9.2) [102]. To evaluate the similarity and difference between the gene modules identified by these methods, we calculated the Jaccard similarity coefficient (referred to here as the module share index) [103], which is measured as the ratio of the intersection to the union of genes between all module pairs.

### Cross-tissue module preservation analysis

To evaluate the preservation of cross-tissue modules in the network, we used the modulePreservation function in the WGCNA R package [104]. Zsummary composite preservation scores were calculated by averaging several preservation statistics generated through many permutations of the raw data, which summarized the evidence that a module is preserved and indicative of module robustness and reproducibility [104]. Generally, modules with Zsummary scores  > 10 were considered strongly preserved (tissue-shared modules), modules with Zsummary scores between 2  and 10 were weakly to moderately preserved, and modules with Zsummary scores < 2 were not preserved (tissue-specific modules) [104,105].

### GWAS and fine-mapping

We performed the single-marker GWAS for five wool traits (MFD, CV, CN, MSL, and GFW), five reproduction traits (GL, LSB, LMWLB, TLWB, and NI), and two growth traits (LW and IBW) in a population of 1639 sheep with genotypes of the 600K arrays (*n* = 504,805 SNPs). For wool traits and LW, we used the adjusted phenotypes (regressing out fixed effects, including flock, birth-year, and month) for GWAS analysis [6]. For reproduction traits and IBW, we used de-regression proof (DRP) as phenotypes.

Genotype imputation was performed using the BEAGLE (v5.1) software [106], and we only considered SNPs with call rate > 90%, minor allele frequency (MAF) > 1%, *P*  _(Hardy–Weinberg equilibrium)_ > 1 × 10^−6^ [6]. We then employed the linear mixed model implemented in GCTA (v1.94.1) [107] software to test for the association of genomic variants with all the complex traits. For each trait, we considered FDR < 0.05 as a threshold to identify the significant SNPs [108].

We constructed the LD matrix for the variants within a 500-kb window flanking each significant SNP (identified in the aforementioned GWAS analysis) using Plink (v1.9) [109]. We identified the causal variants linked to the significant SNPs by analyzing the summary statistics of GWAS using the *susie_rss* function in the susieR R package (v0.12.35) [110]. Subsequently, the causal effect was evaluated by computing PIP. Locus plots with gene annotations were plotted using the locuszoomr R package (v0.3.5).

### Enrichment analysis of GWAS signals in tissue- and development-specific genes

We applied the sum-based marker-set test approach implemented in the R package QGG [111] to identify whether the GWAS signals were significantly enriched in a gene set, including tissue-specific genes, development-specific genes, and co-expression modules:


$$T_{\mathrm{sum}}=\sum_{i=1}^{m_{g}}b^{2}$$

where *T*_sum_ is the summary statistics of a gene set, $m_{g}$ is the number of SNPs within the gene set, and b is the SNP effect from single-marker GWAS. We considered SNPs within 20 kb upstream and downstream of the gene body to cover the *cis*-regulatory regions of genes. To obtain an empirical *P* value for the association of a gene set with a complex trait, we employed a 10,000 times permutation strategy and then applied a one-tailed test of the proportion of random summary statistics greater than that observed, as described previously [8,27]. We then corrected *P* values for multiple testing using the FDR method and considered FDR < 0.05 significant.

## Ethical statement

All animal experiments were carried out in accordance with the ARRIVE Guidelines and the Regulations for the Administration of Affairs Concerning Experimental Animals (revised in March 2017) approved by the State Council of the People’s Republic of China, and also approved by the Animal Ethical and Welfare Committee of Xinjiang Academy of Animal Sciences, China (Approval No. 2019009). The Australian animal experiments were conducted at the University of New England, Armidale, Australia and approved by the University of New England Animal Ethics Committee (Approval No. AEC no14-041) [112]. The pregnant ewes were housed indoors for a 7-day “settling-in period” before the experiment, during which they had ad libitum access to feed and water. The ewes were not fed the night before they were euthanized by electrocution followed by exsanguination.

## Supplementary Material

qzaf020_Supplementary_Data

## Data Availability

The raw sequence data reported in this study have been deposited in the Genome Sequence Archive [113] at the National Genomics Data Center (NGDC), China National Center for Bioinformation (CNCB) (GSA: CRA020496), and are publicly accessible at https://ngdc.cncb.ac.cn/gsa.

## CRediT author statement

**Bingru Zhao:** Data curation, Formal analysis, Investigation, Writing – original draft, Writing – review & editing. **Hanpeng Luo:** Software, Formal analysis, Data curation. **Xuefeng Fu:** Validation, Investigation, Resources. **Guoming Zhang:** Visualization, Investigation. **Emily L. Clark:** Data curation, Writing – original draft & editing. **Feng Wang:** Validation, Investigation. **Brian Paul Dalrymple:** Data curation, Writing – review & editing. **V. Hutton Oddy:** Data curation, Writing – review & editing. **Philip E. Vercoe:** Data curation, Writing – review & editing. **Cuiling Wu:** Resources. **George E. Liu:** Resources. **Cong-jun Li:** Resources. **Ruidong Xiang:** Formal analysis, Writing – review & editing, Funding acquisition. **Kechuan Tian:** Conceptualization, Writing – review & editing, Funding acquisition, Project administration. **Yanli Zhang:** Conceptualization, Formal analysis, Writing – review & editing, Funding acquisition, Project administration. **Lingzhao Fang:** Conceptualization, Methodology, Formal analysis, Investigation, Writing – review & editing, Project administration. All authors have read and approved the final manuscript.

## ORCID

0009-0004-0140-8654 (Bingru Zhao)

0000-0001-6211-3834 (Hanpeng Luo)

0000-0001-6308-1109 (Xuefeng Fu)

0000-0002-4729-518X (Guoming Zhang)

0000-0002-9550-7407 (Emily L. Clark)

0000-0002-4965-836X (Feng Wang)

0000-0003-3891-5233 (Brian Paul Dalrymple)

0000-0003-1783-1049 (V. Hutton oddy)

0000-0002-3061-1908 (Philip E. Vercoe)

0000-0001-5863-5744 (Cuiling Wu)

0000-0003-0192-6705 (George E. Liu)

0000-0003-1389-9820 (Cong-jun Li)

0000-0002-1584-7605 (Ruidong Xiang)

0000-0002-4964-0629 (Kechuan Tian)

0000-0002-7901-7774 (Yanli Zhang)

0000-0003-1103-3679 (Lingzhao Fang)
