# Supplementary material for: A Developmental Gene Expression Atlas Reveals Novel Biological Basis of Complex Phenotypes in Sheep
Source: Genomics Proteomics Bioinformatics. 2025 Mar 4;23(1):qzaf020. doi: 10.1093/gpbjnl/qzaf020 (PMC12228968; doi:10.1093/gpbjnl/qzaf020)
Supplement: qzaf020_Supplementary_Data [file qzaf020_supplementary_data.zip › supplementary_material_captions.docx]

**Supplementary material**

**Figure S1 Data summary of the sheep developmental transcriptome**

**A.** The Spearman’s correlation of gene expression (transcripts per million (TPM)) among three transcript quantification tools: Kallisto, Strigtie and Salmon. **B.** Distribution of uniquely mapped ratio across all samples. **C.** Distribution of numbers of clean reads across all samples. **D.** Number of expressed genes (Transcripts per Million, TPM > 0.1) increases rapidly with the increasing number of clean reads across all 1557 samples; 60% Ensembl genes were detected at 10 million reads, reaching a plateau at 50 million reads. The black line is the smoothed curve fitted by a generalized additive model using the geom_smooth function from ggplot2 (v3.4.1) in R (v4.2.2). The shaded area around the lines represents the 95% confidence interval for the fitted values (the line). **E.** BioProject distribution of samples in the 1464 high-quality RNA-seq datasets (uniquely mapped ratio > 0.6 & clean reads > 1000,000). **F.** Breed distribution of samples. **G.** Sex distribution of samples. **H.** Distribution of numbers of developmental stages across all tissues. **I.** In the percentage of RNA-seq with single or paired reads, 99.4% of samples were paired-end sequencing, and only 0.6% of samples were single-end sequencing.

**Figure S2 Characteristics of the sheep developmental Gene Expression Atlas (dGEA)**

**A.** Number of expressed genes (Transcripts per Million, TPM > 0.1) increases with the increasing number of clean reads across all 1464 samples; 60% of Ensembl genes were detected at 10 million reads, reaching a plateau at 50 million reads. **B.** The correlation between the number of expressed genes (TPM > 0.1) and clean reads for each tissue category. For tissues with multiple samples, we calculate the median of expressed genes among all these samples. **C.** Comparison of the tissue specificity metric reflected by the TAU score of five transcript types**. D.** and **E.** The distribution of gene expression across the number of tissues and stages, respectively. The expression (TPM) of genes was averaged in a given number of tissues or stages. **F.** Distribution of expressed genes (TPM > 0.1) ratio with the different gene types (protein-coding genes (PCGs), long non-coding RNAs (lncRNAs), microRNA (miRNAs), mitochondrial RNA (mtRNA), pseudogenes and others) across all 51 tissues. **G.** Transcriptome complexity. At the bottom, the cumulative distribution of the average fraction of total transcription contributed by genes is shown, with genes sorted from most to least expressed in each tissue on the x-axis. The lines represent the mean values across samples of the same tissue, while the lighter-colored surfaces surrounding the mean indicate dispersion calculated as the standard deviation divided by the cumulative sum of all means. At the top, the hundred most expressed genes are illustrated, indicating their biological type and relative contribution to total transcription. The height of the bars corresponds to the fraction that these genes contribute to total transcription, providing a visual representation of their significance.

**Figure S3 Sample clustering based on gene expression and alternative splicing profiles**

**A.** Principal component analysis (PCA) of samples based on the scaled expression (*i.e.*, Log_2_ (TPM + 0.25)) of 6000 genes with the highest expression variance across 20 tissue categories. **B.** and **C.** PCA of samples based on the alternative splicing (reflected by percent spliced in, PSI) of 6000 spliced introns with the highest variance across 51 tissues and 20 tissue categories, respectively. **D.** Correlation clustering of samples based on distances between gene expression levels of samples measured by Pearson’s correlation.

**Figure S4 Principal component analysis (PCA) of samples based on gene expression in each tissue across developmental stages.**

This includes early-prenatal (embryo, E16-E70), late-prenatal (E80-E135), neonate (postnatal, P0-P8), lamb (week3-month6), juvenile (month7-year1.5), adult (year, Y2-Y4), and elderly (Y7).

**Figure S5 Clustering analysis of RNA-seq samples reveals germ layer gene expression patterns**

**A.** Hierarchical clustering of 51 tissues based on the median expression of 6000 genes with the highest expression variance across samples, representing embryo, endodermal, mesodermal and ectodermal lineages. **B.**−**D.** Pearson’s correlation of gene expression in tissues derived from endodermal, mesodermal, and ectodermal lineages, respectively.

**Figure S6 The normalized consensus scores of significantly (FDR < 0.05) enriched Gene Ontology (GO) terms for all upregulated tissue-specific genes by the gene set enrichment analysis (GSEA)**

**Figure S7 Tissue specificity of alternative splicing**

**A.** Median expression profile of tissue-specific intron splicing across tissues. Each row represents a gene, and each column represents an individual tissue. Color represents median PSI of intron splicing among samples in a tissue. **B.** Significant terms (*P* < 0.05) of Gene Ontology (GO) enrichment for genes with spliced introns based on the hypergeometric test across 51 tissues.

**Figure S8 Number of stage-specific genes across developmental stages for each of 20 tissues**

**A.** Number of all stage-specific genes. **B.** Number of upregulated stage-specific genes. **C.** Number of downregulated stage-specific genes. Each violin represents a developmental stage, and each dot represents a tissue. The color of tissues and developmental stages corresponds to that shown in Figure 1.

**Figure S9 Features of stage-specific genes in major tissues**

**A.** The panels for heart from left to right illustrate the distribution of different gene types (protein-coding genes (PCGs), long non-coding RNAs (lncRNAs), microRNA (miRNAs), mitochondrial RNA (mtRNA), pseudogenes and others) among stage-specific genes; the expression level (Log_2_ (TPM + 1)) of stage-specific genes across developmental stages; the biological processes enriched with stage-specific genes; motifs of transcriptional factors (TFs) that were significantly enriched in promoters of stage-specific PCGs, respectively. **B.−E.** Similar to A but for lung, kidney, lymph node, and spleen, respectively.

**Figure S10 Features of stage-specific genes in major tissues**

**A.** The panels for lleum from left to right illustrate the distribution of different gene types (protein-coding genes (PCGs), long non-coding RNAs (lncRNAs), microRNA (miRNAs), mitochondrial RNA (mtRNA), pseudogenes and others) among stage-specific genes; the expression level [Log_2_ (TPM + 1)] of stage-specific genes across developmental stages; the biological processes enriched with stage-specific genes; motifs of transcriptional factors (TFs) that were significantly enriched in promoters of stage-specific PCGs, respectively. **B.−E.** Similar to A but for omasum, pylorus, tonsil/tongue, and thymus, respectively.

**Figure S11 Features of stage-specific genes in GI tract tissues**

**A.** The panels for jejunum from left to right illustrate the distribution of different gene types (protein-coding genes (PCGs), long non-coding RNAs (lncRNAs), microRNA (miRNAs), mitochondrial RNA (mtRNA), pseudogenes and others) among stage-specific genes; the expression level [Log_2_ (TPM + 1)] of stage-specific genes across developmental stages; the biological processes enriched with stage-specific genes; motifs of transcriptional factors (TFs) that were significantly enriched in promoters of stage-specific PCGs, respectively. **B.−E.** similar to A but for reticulum, cecum, colon, and duodenum, respectively.

**Figure S12 Trajectory clustering of all tissue-specific genes in all 20 tissues across four developmental stages**

These trajectories were grouped into eight clusters baesd on the soft-clustering approach (c-means). Four clusters are displayed as examples in Figure 5A, others are showed here. Percentage of tissue-specific genes are shown next to each cluster. The strongest enriched gene ontology (GO) term (*P* < 0.05) of biological processes for each cluster is listed the below.

**Figure S13**  **Clustering of stage-specific genes based on the soft-clustering approach (c-means) in all developmental stages for each major tissues**

Average maximum-normalized expression values for all stage-specific genes in each of the clusters generated by mFuzz. Numbers in each top panel indicate the number of genes grouped in that cluster, and polygons represents ± 1 standard deviation. The strongest enriched gene ontology (GO) term (*P* < 0.05) of biological processes for each cluster in corresponding tissues are listed the below.

**Figure S14 Clustering of stage-specific genes based on the soft-clustering approach (c-means) in all developmental stages for immune tissues**

Average maximum-normalized expression values for all stage-specific genes in each of the clusters generated by mFuzz. Numbers in each top panel indicate the number of genes grouped in that cluster, and polygons represents ± 1 standard deviation. The strongest enriched gene ontology (GO) term (*P* < 0.05) of biological processes for each cluster in corresponding tissues are listed below.

**Figure S15 Clustering of stage-specific genes based on the soft-clustering approach (c-means) in all developmental stages for gastrointestinal (GI) tract** Average maximum-normalized expression values for all stage-specific genes in each of the clusters generated by mFuzz. Numbers in each top panel indicate the number of genes grouped in that cluster, and polygons represents ± 1 standard deviation. The strongest enriched gene ontology (GO) term (*P* < 0.05) of biological processes for each cluster in corresponding tissues are listed below.

**Figure S16 Heatmap shows the normalized gene expression of the NR1D1 gene in 13 tissues across developmental stages**

**Figure S17 Gene module detection and comparison across multi-tissue and single-tissue co-expression analyses**

**A.** Number of gene modules detected from the whole dataset (integrated approach) by five gene co-expression approaches. **B.** and **C.** Number of genes detected from modules on the whole dataset (multi-tissue approach) and each individual tissue (separated approach) by multiple gene co-expression approaches. The modules detected from WGCNA, GWENA and CEMiTool are non-overlapping, while ICA, MEGENA and PEER could define larger modules that share the same set of genes. **D.** The module share index (the intersection/union of genes between all module pairs) showed some pairs from the same tissue share the same genes. **E.** Module sharing index between WGCNA and the remaining five methods. **F**, Module sharing index between WGCNA and the remaining five methods in each of the 20 tissues. **G.** and **H.** Distribution of unannotated genes across on the whole dataset (integrated approach) and each individual tissue (separated approach) by multiple gene co-expression approaches.

**Figure S18 Cross-tissue preservation scores (Zsummary scores) of gene co-expression modules**

The x-axis represents the number of genes in a module.

**Figure S19** **The weighted gene co-expression network analysis (WGCNA)**

**A.** Correlations between gene modules and developmental stages in liver. The statistical significance of module-developmental stage relationship is corrected for multiple testing using the FDR method. The yellow stars denote FDR < 0.05. Each cell contains the correlation and the corresponding FDR value in bracket. The heatmap in the middle shows the top enriched significant GO terms (biological process; BP) for significantly related modules, and the expression pattern of the top representative module-enriched TFs and sequence motif are shown on the right. **B.** Similar to A, but for kidney.

**Figure S20 Expression patterns of genes associated with Mendelian trait/disorder in sheep specifically expressed in tissues across developmental stages**

The Mendelian trait/disorder associated with each gene is listed next to it.

**Figure S21 Tracing specific gene sets across tissues and time to interpret GO terms**

**A.** Expression levels of the 12 genes enriched in GO term of lipid metabolic process. The gene expression levels are standardized as transcripts per million (TPM). The y-axis represents the 12 genes, and the x-axis represents the 51 tissues. **B.** Expression patterns of 12 genes in adipose across developmental stages.

**Figure S22 Enrichment of GWAS signals in genes with specific splicing patterns across tissues and developmental stages**

**A.** GWAS signal enrichment results of 12 complex traits in 51 tissue-specific introns. The color corresponds to the enrichment degree (*i.e.*, −Log_10_ FDR), which was computed by a sum-based GWAS signal enrichment analysis based on the top 5% tissue-specific introns and a 20-kb extension, where (*) means FDR < 0.05. The x-axis represents 12 economic traits (MFD, mean fibre diameter; CV, coefficient of variation of the fibre diameter; CN, crimp number; MSL, mean staple length; GFW, greasy fleece weight; GL, gestation length; LSB, litter size at birth; LMWLB, litter mean weight per lamb born; TLWB, total litter weight at birth; NI, number of mating pregnancy; IBW, individual birthweight; LW, live weight). **B.** The GWAS signal enrichments (−Log_10_ *P* value) degree was compared using stage-specific genes across different developmental stages for each tissue. To compare the significance of GWAS enrichment degree across different developmental stages, we conducted ANOVA test, and the late-prenatal was selected as the target stage for pairwise comparisons with other stages, where (**) means *P* value < 0.01. **C** Similar to **A**, but for stage-specific genes in rumen, reticulum, abomasum, lymph node, skin, duodenum, jejunum, cecum, colon, muscle, liver, respectively.

**Table S1 The metadata of the sheep developmental Gene Expression Atlas (dGEA)**

**Table S2 The gene expression estimates as transcripts per million (TPM) relating to all RNA-seq samples**

**Table S3 Enrichment analyses of GWAS signals using tissue-specific genes, stage-specific genes, and gene clusters for 12 complex traits in sheep**

**Table S4 Enrichment analyses of GWAS signals using tissue- and stage-specific splicing for 12 complex traits in sheep**

**Table S5 Significant SNPs and candidate genes identified for wool, reproduction, and growth traits in sheep**

**Table S6 Fine-mapping for significant SNPs associated with wool, reproduction, and growth traits in sheep**
