# Supplementary figures and images for: A Developmental Gene Expression Atlas Reveals Novel Biological Basis of Complex Phenotypes in Sheep
Source: Genomics Proteomics Bioinformatics. 2025 Mar 4;23(1):qzaf020. doi: 10.1093/gpbjnl/qzaf020 (PMC12228968; doi:10.1093/gpbjnl/qzaf020)

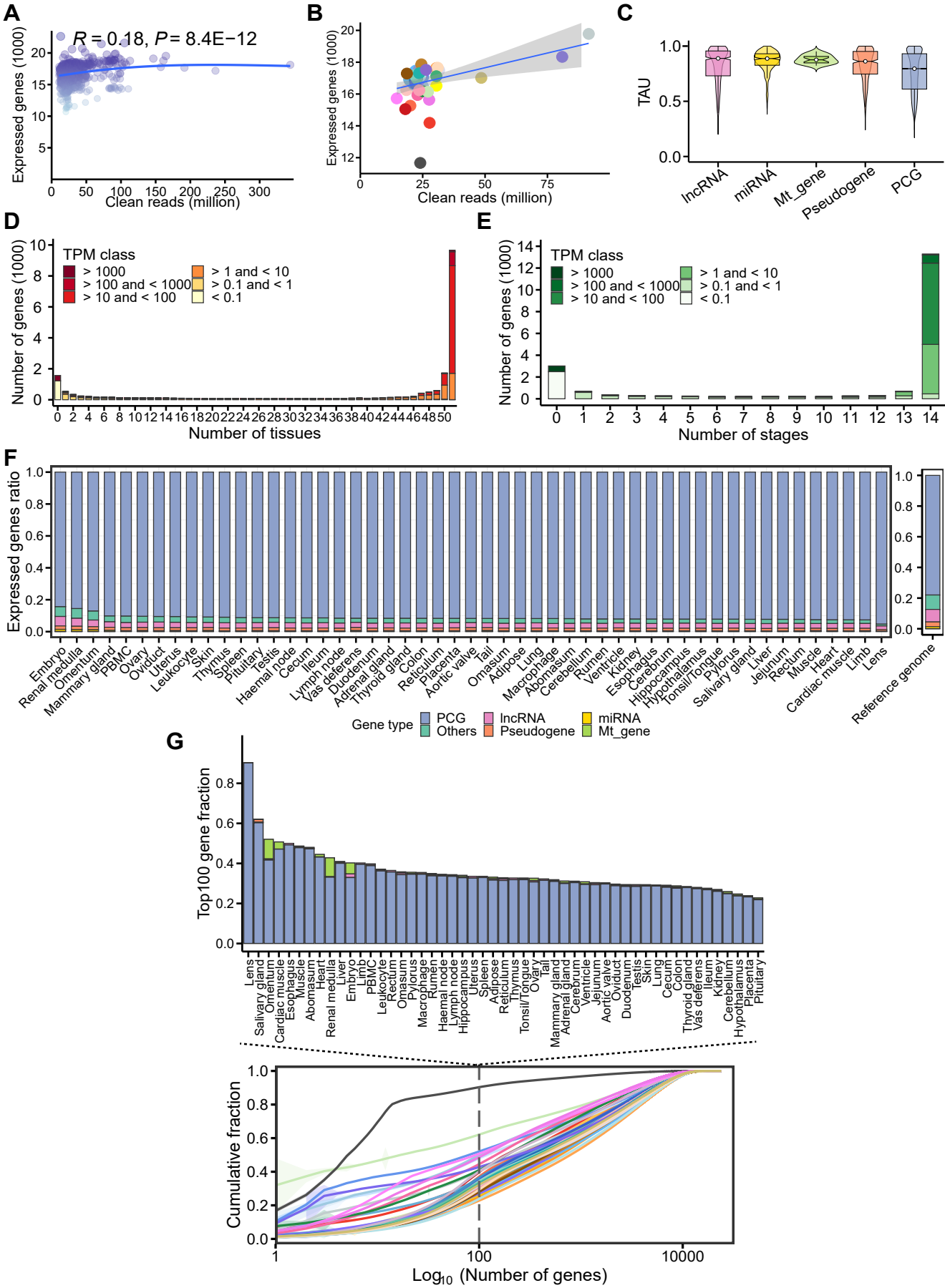

Supplement: qzaf020_Supplementary_Data [file qzaf020_supplementary_data.zip › Figure_S2.pdf]

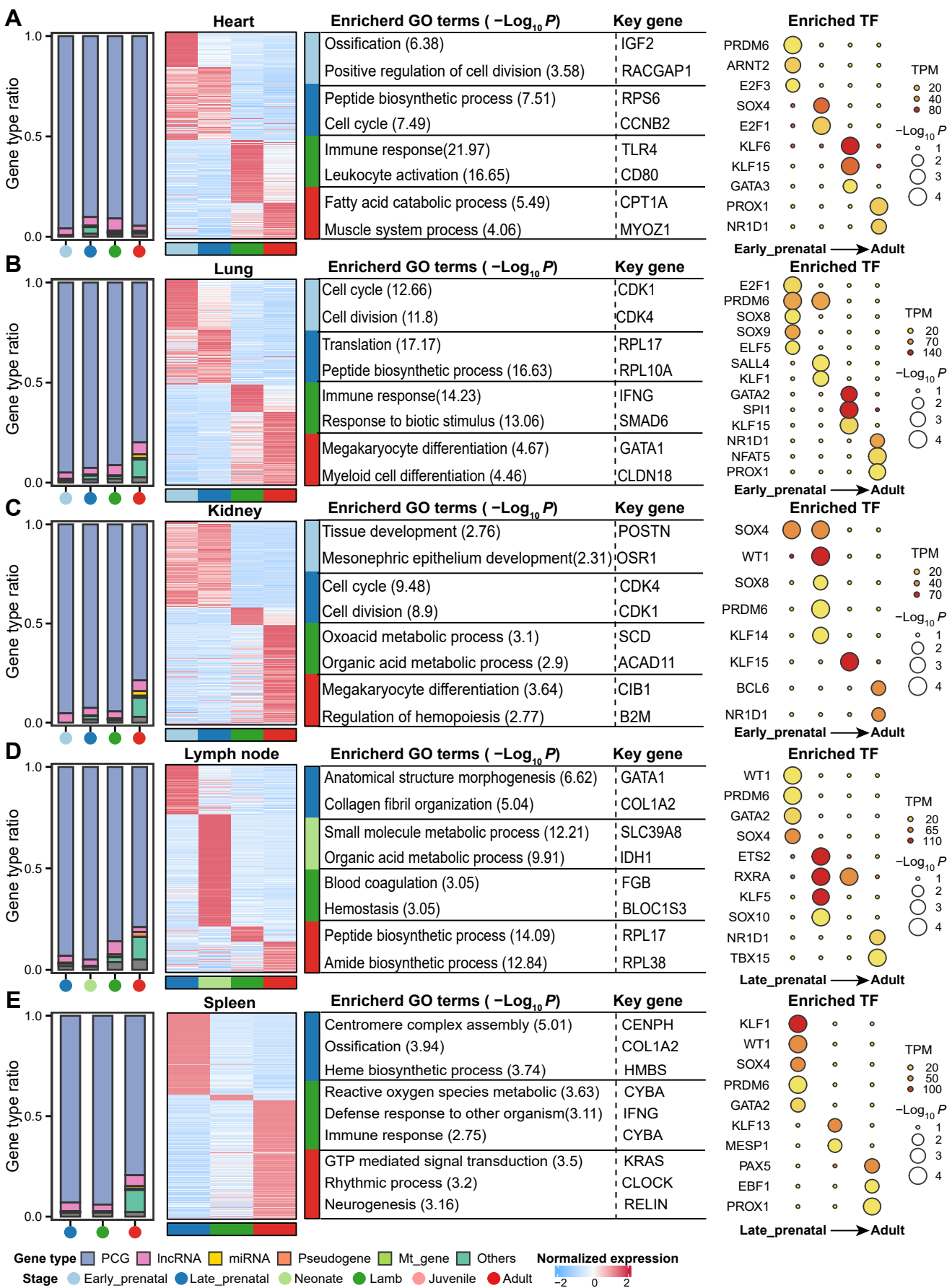

Supplement: qzaf020_Supplementary_Data [file qzaf020_supplementary_data.zip › Figure_S9.pdf]

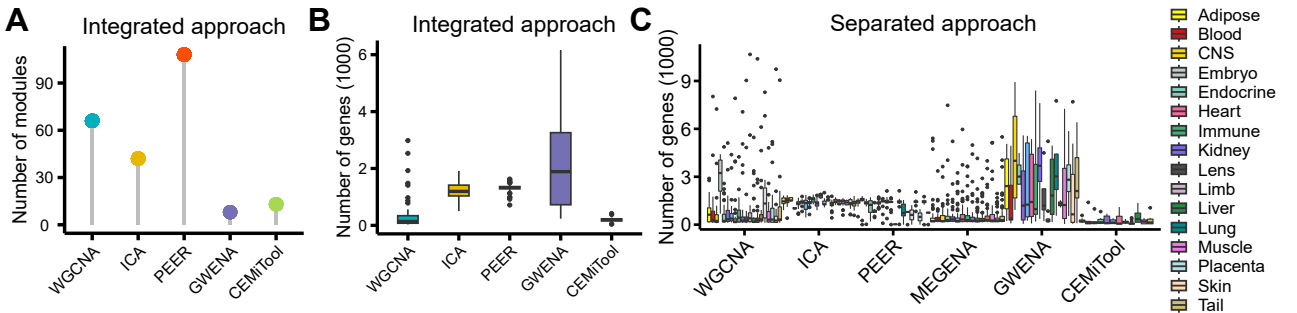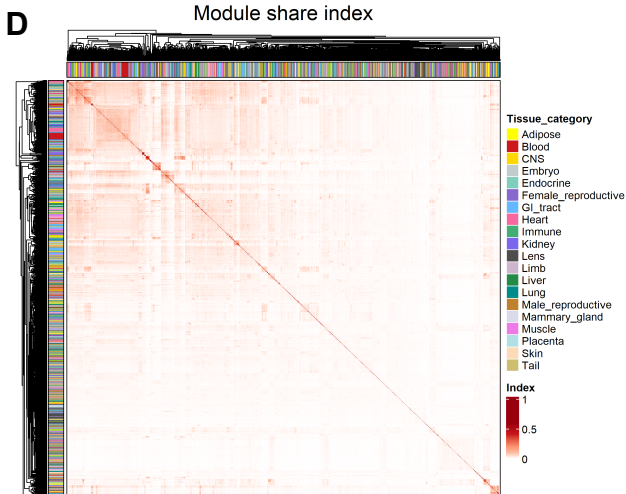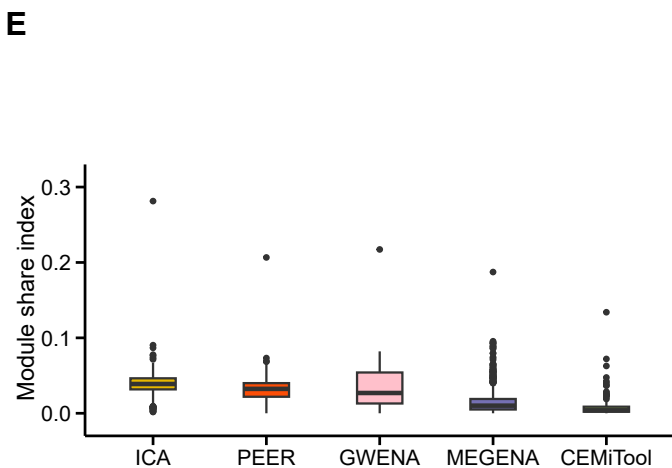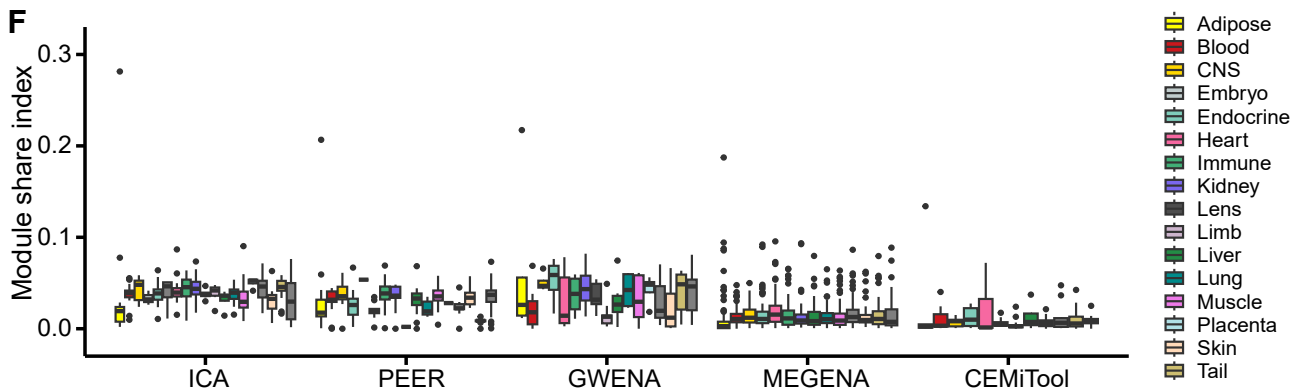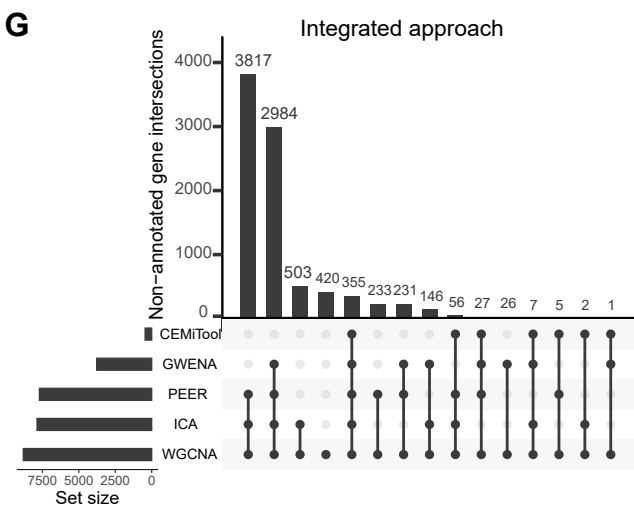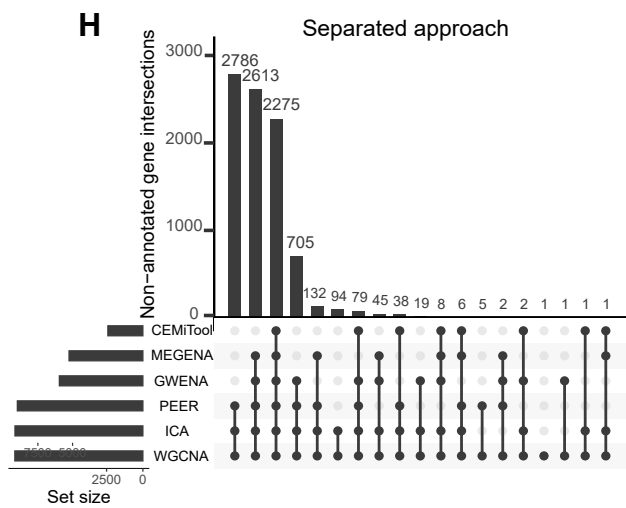

Supplement: qzaf020_Supplementary_Data [file qzaf020_supplementary_data.zip › Figure_S17.pdf]

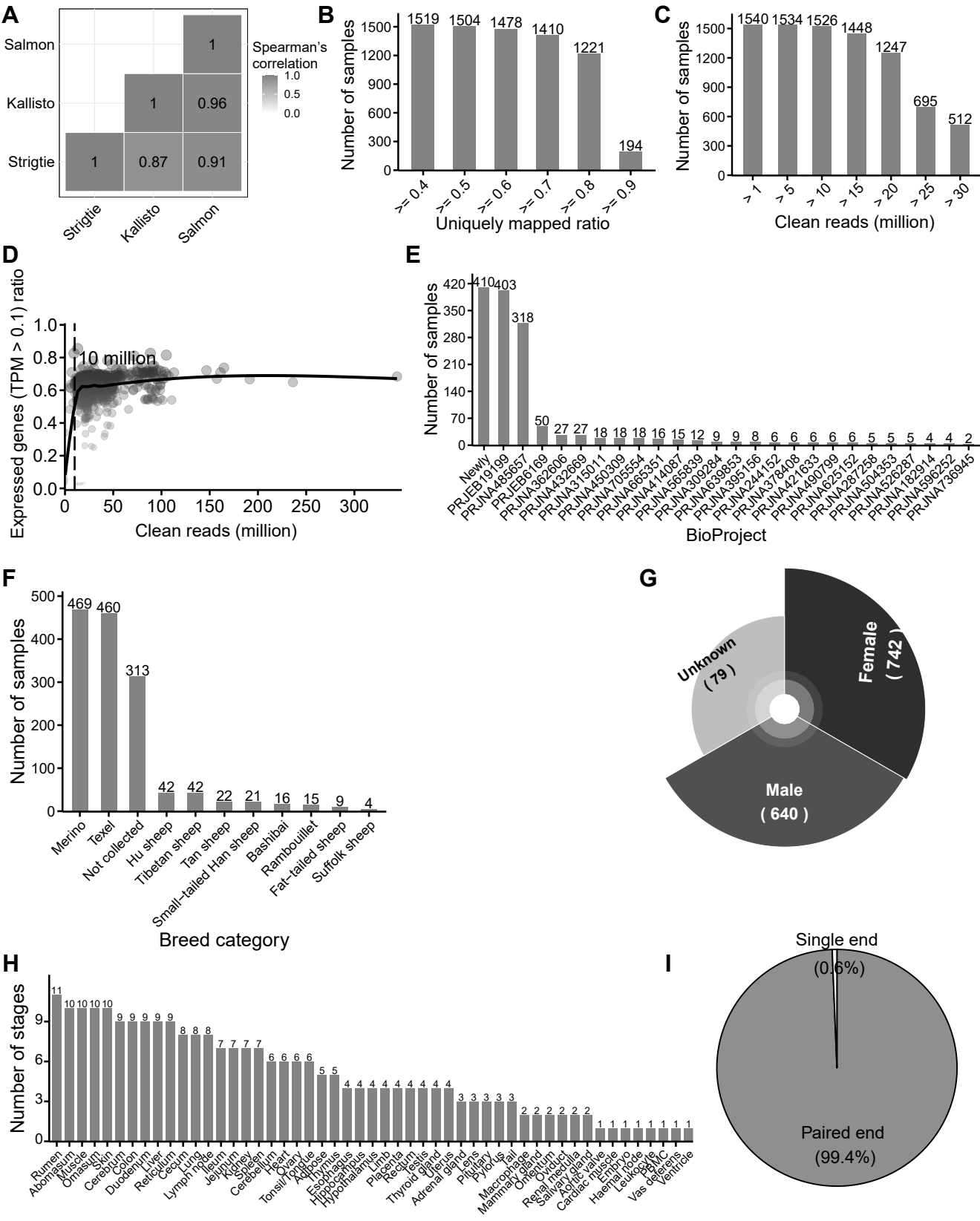

Supplement: qzaf020_Supplementary_Data [file qzaf020_supplementary_data.zip › Figure_S1.pdf]

Preservation Zsummary

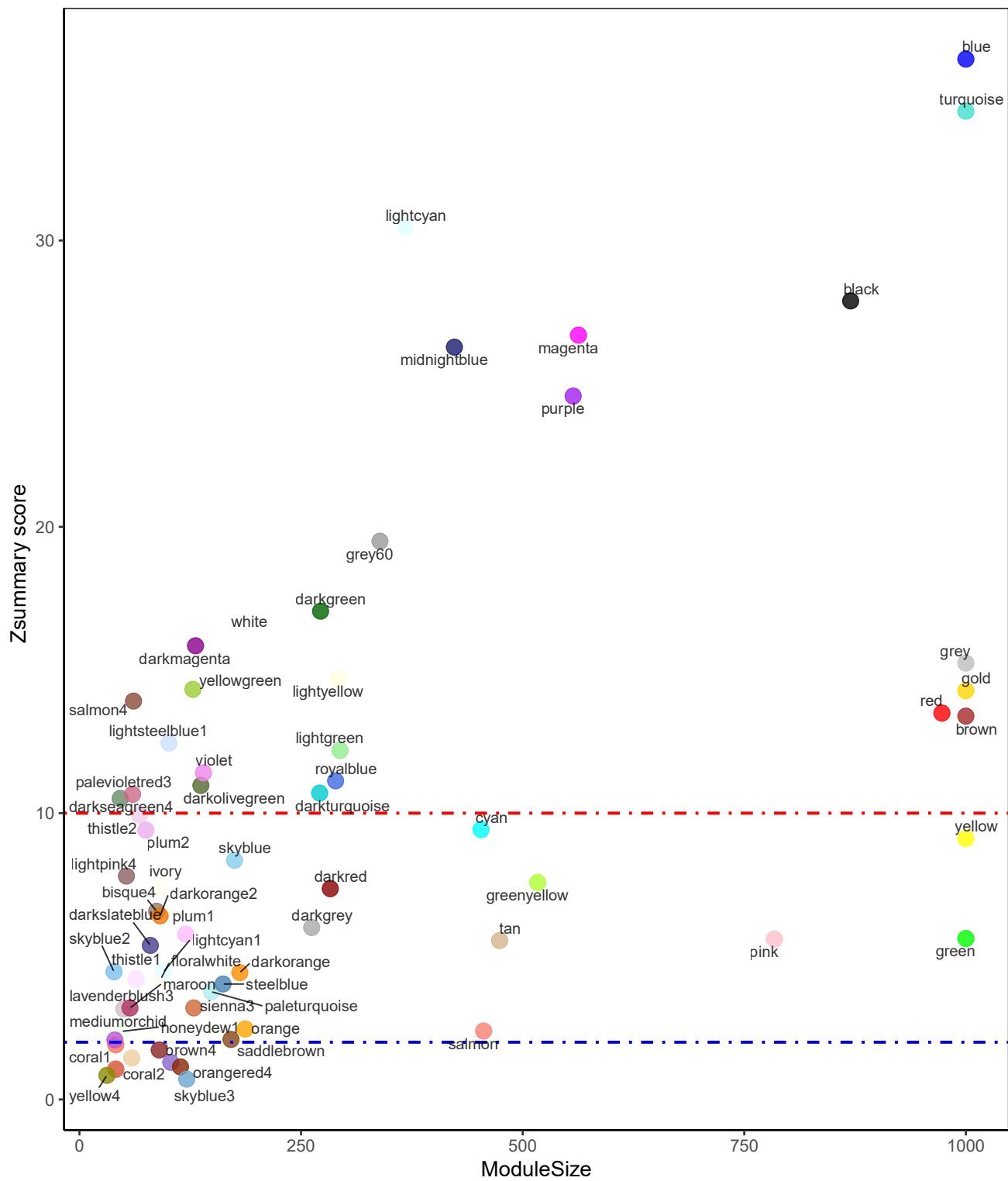

Supplement: qzaf020_Supplementary_Data [file qzaf020_supplementary_data.zip › Figure_S18.pdf]

# NR1D1

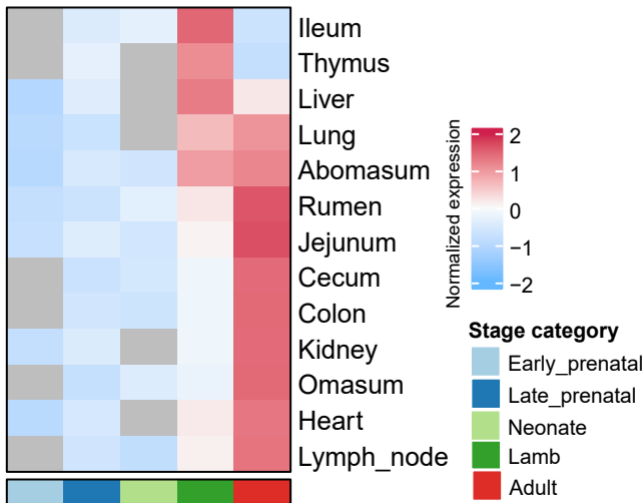

Supplement: qzaf020_Supplementary_Data [file qzaf020_supplementary_data.zip › Figure_S16.pdf]

# Raw gene expression

PC2

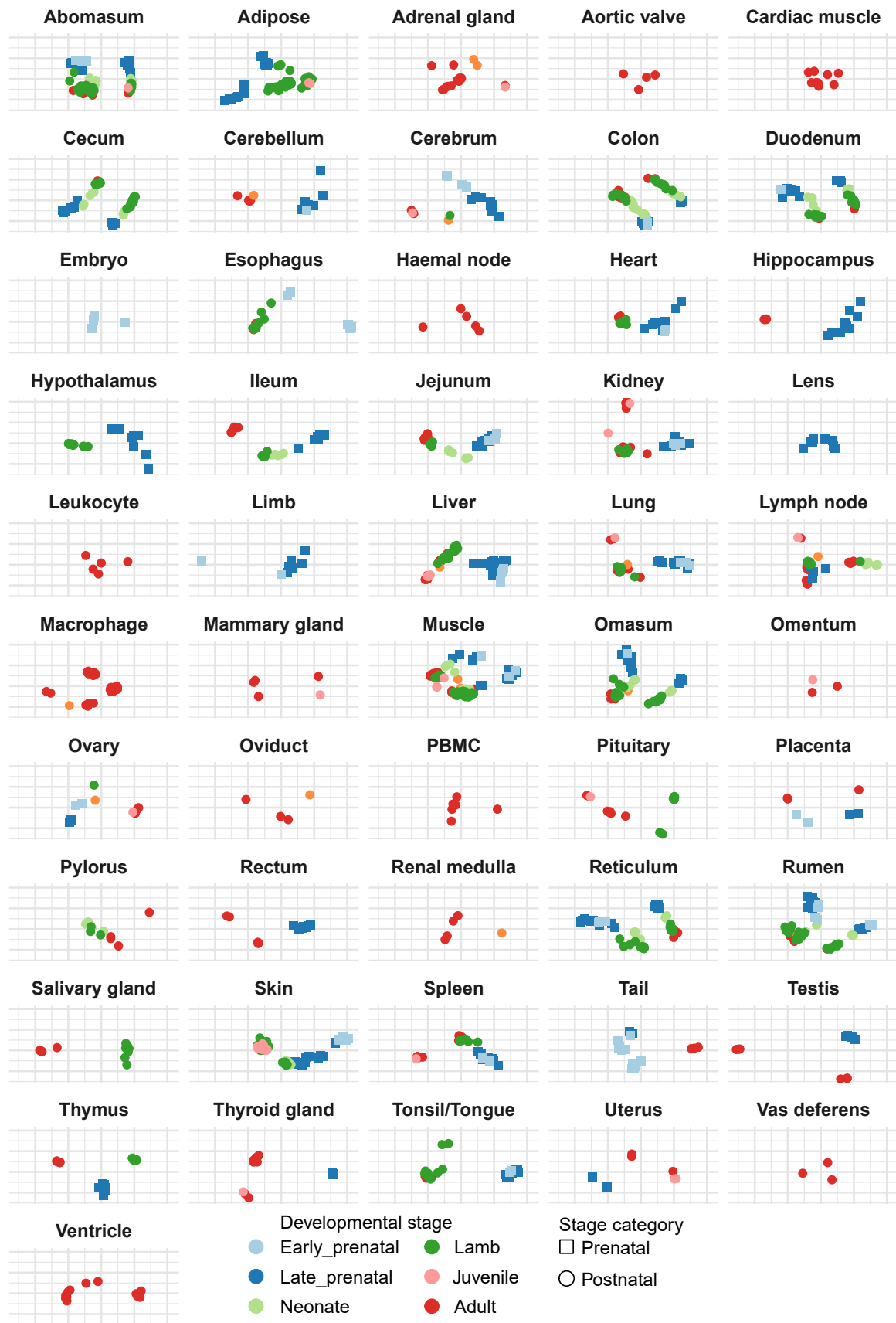

PC1

Supplement: qzaf020_Supplementary_Data [file qzaf020_supplementary_data.zip › Figure_S4.pdf]

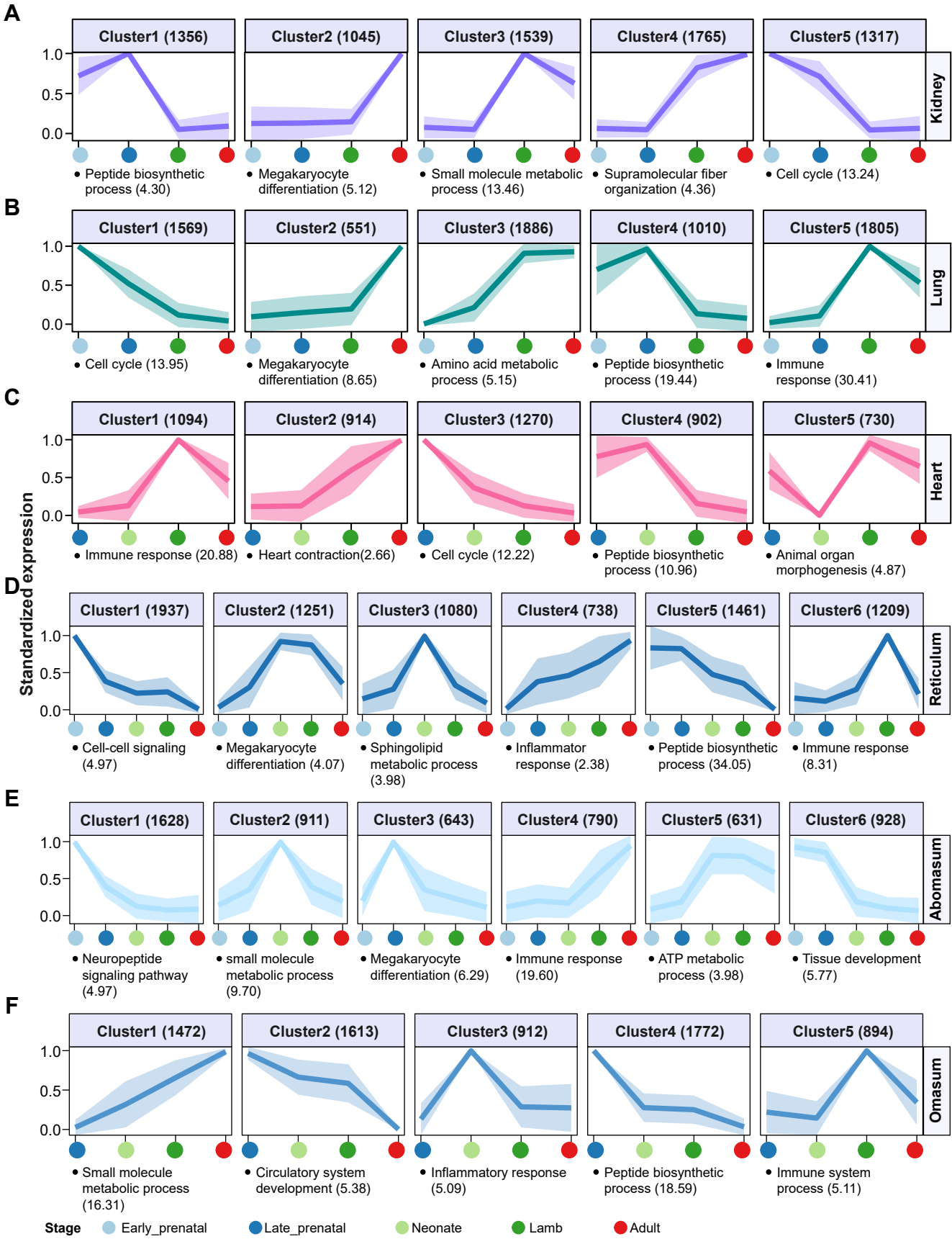

Supplement: qzaf020_Supplementary_Data [file qzaf020_supplementary_data.zip › Figure_S13.pdf]

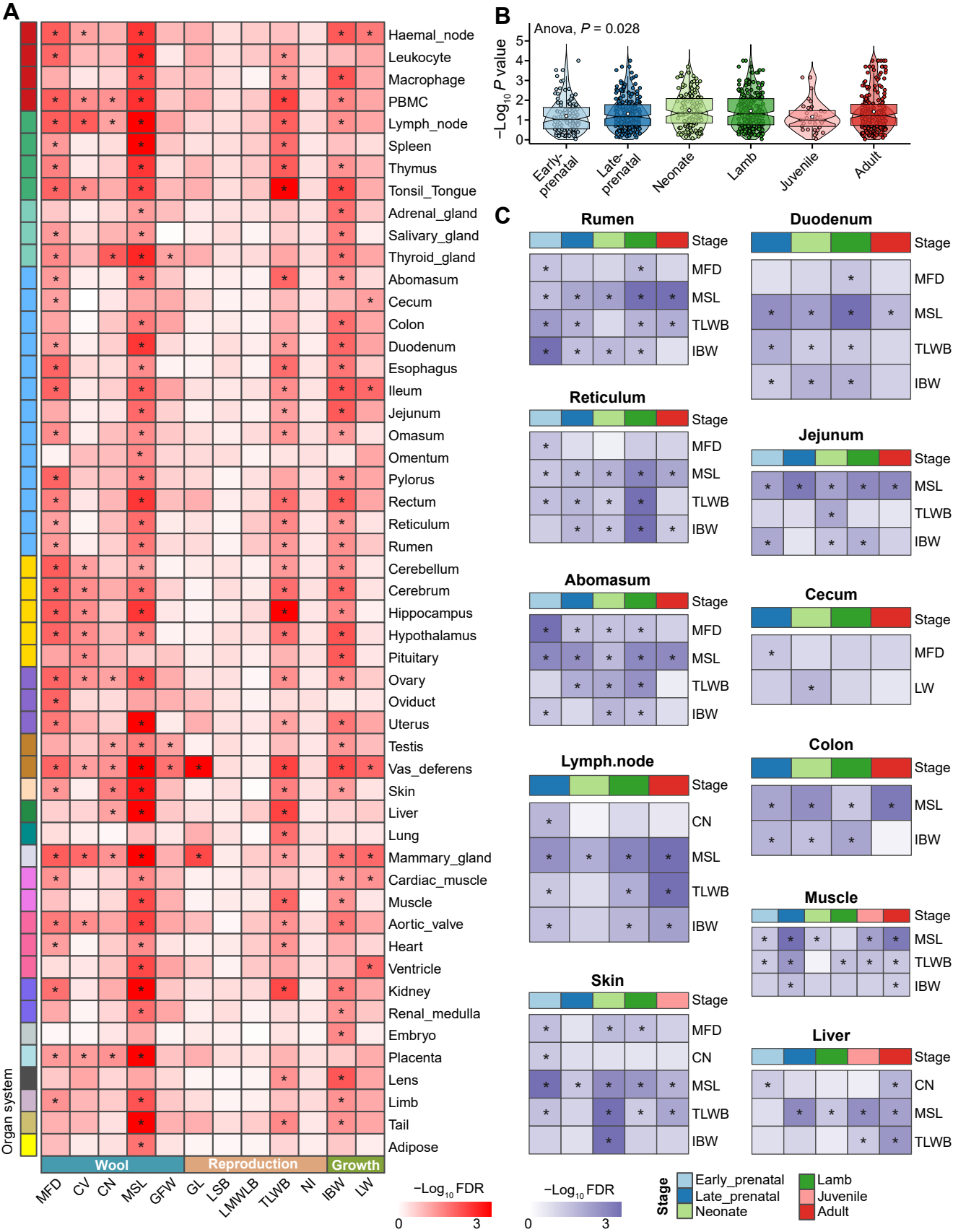

Supplement: qzaf020_Supplementary_Data [file qzaf020_supplementary_data.zip › Figure_S22.pdf]

**A****GO term: lipid metabolic process**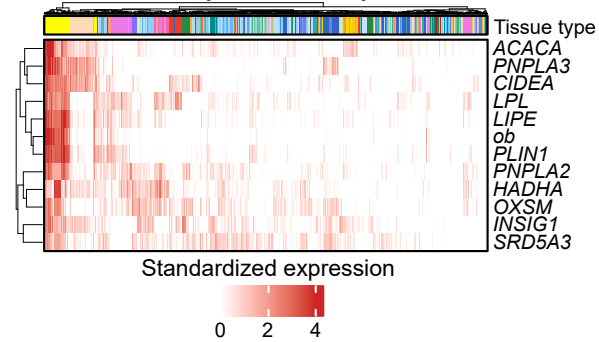**B**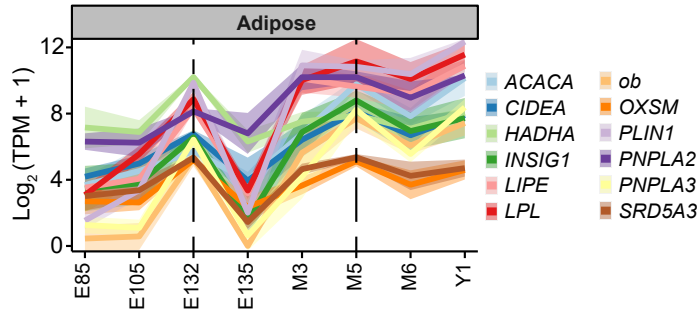

Supplement: qzaf020_Supplementary_Data [file qzaf020_supplementary_data.zip › Figure_S21.pdf]

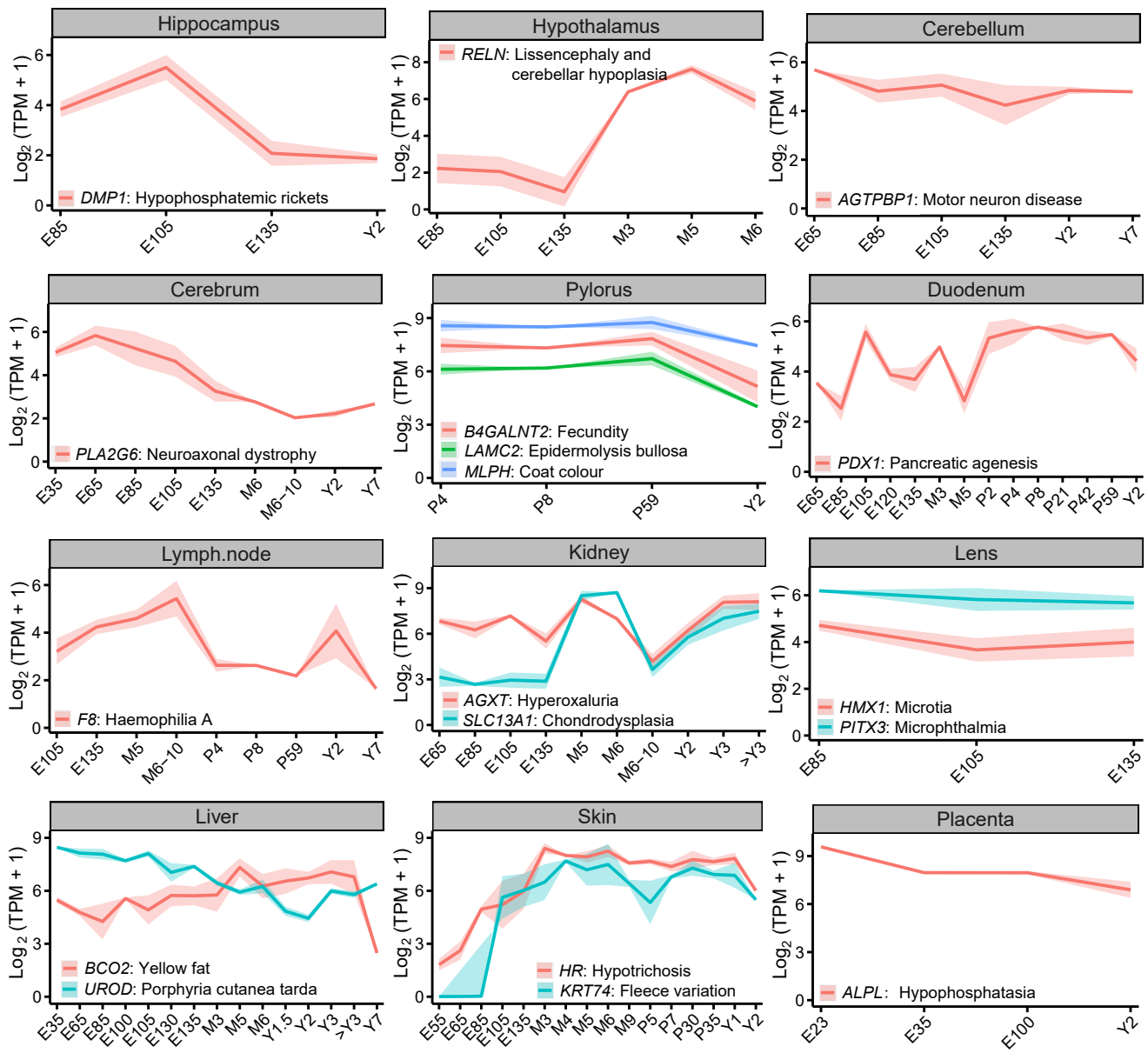

Supplement: qzaf020_Supplementary_Data [file qzaf020_supplementary_data.zip › Figure_S20.pdf]

Standardized expression

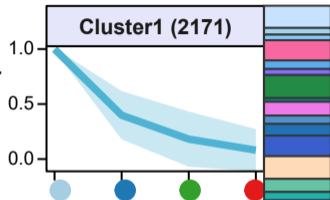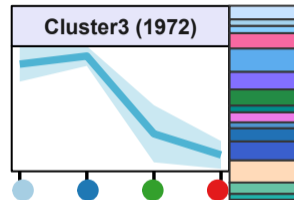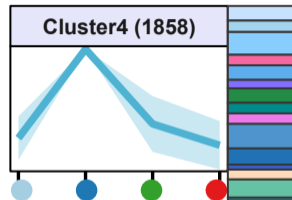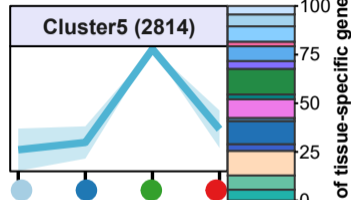

Percentage of tissue-specific genes (%)

Supplement: qzaf020_Supplementary_Data [file qzaf020_supplementary_data.zip › Figure_S12.pdf]

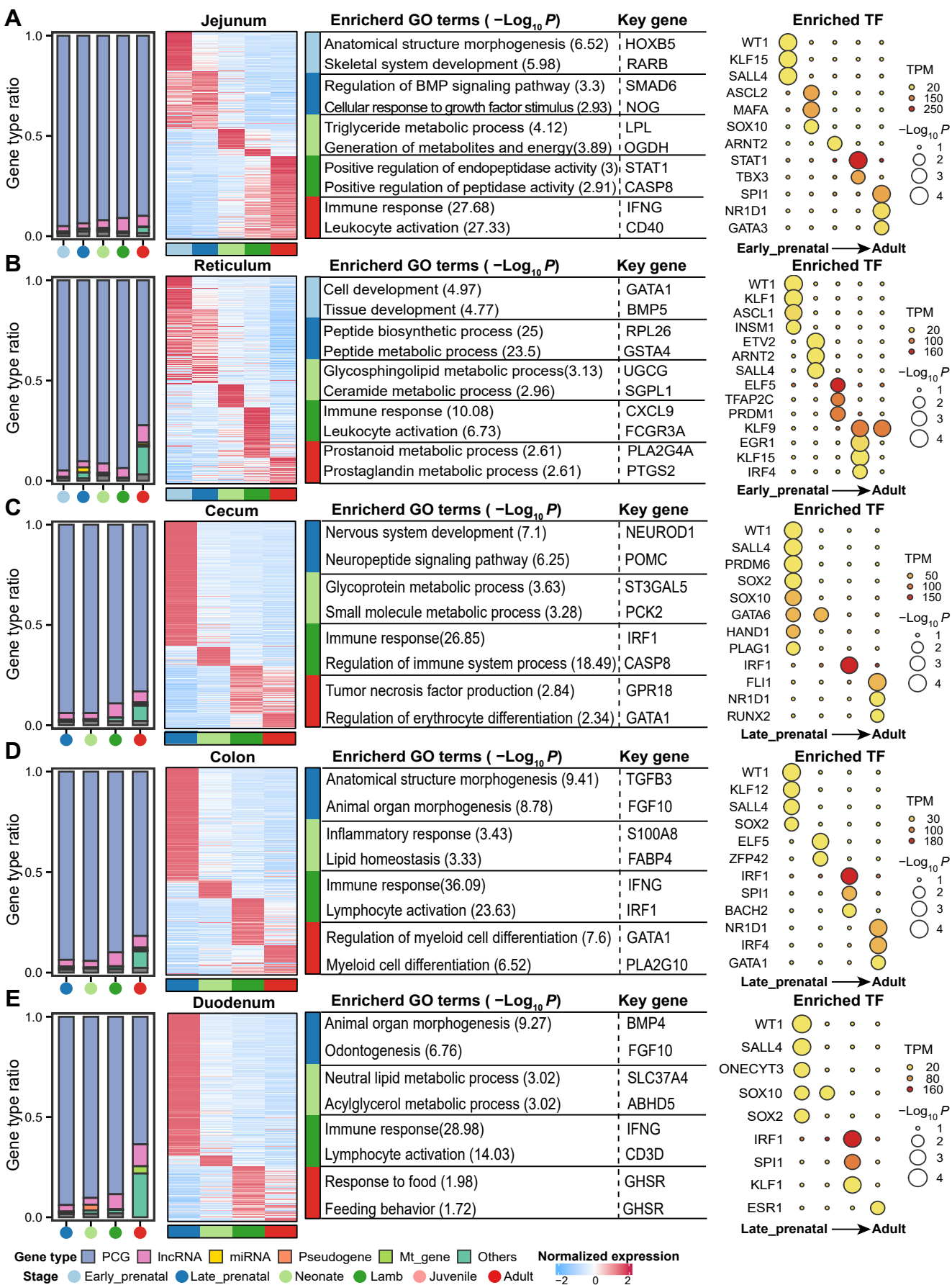

Supplement: qzaf020_Supplementary_Data [file qzaf020_supplementary_data.zip › Figure_S11.pdf]

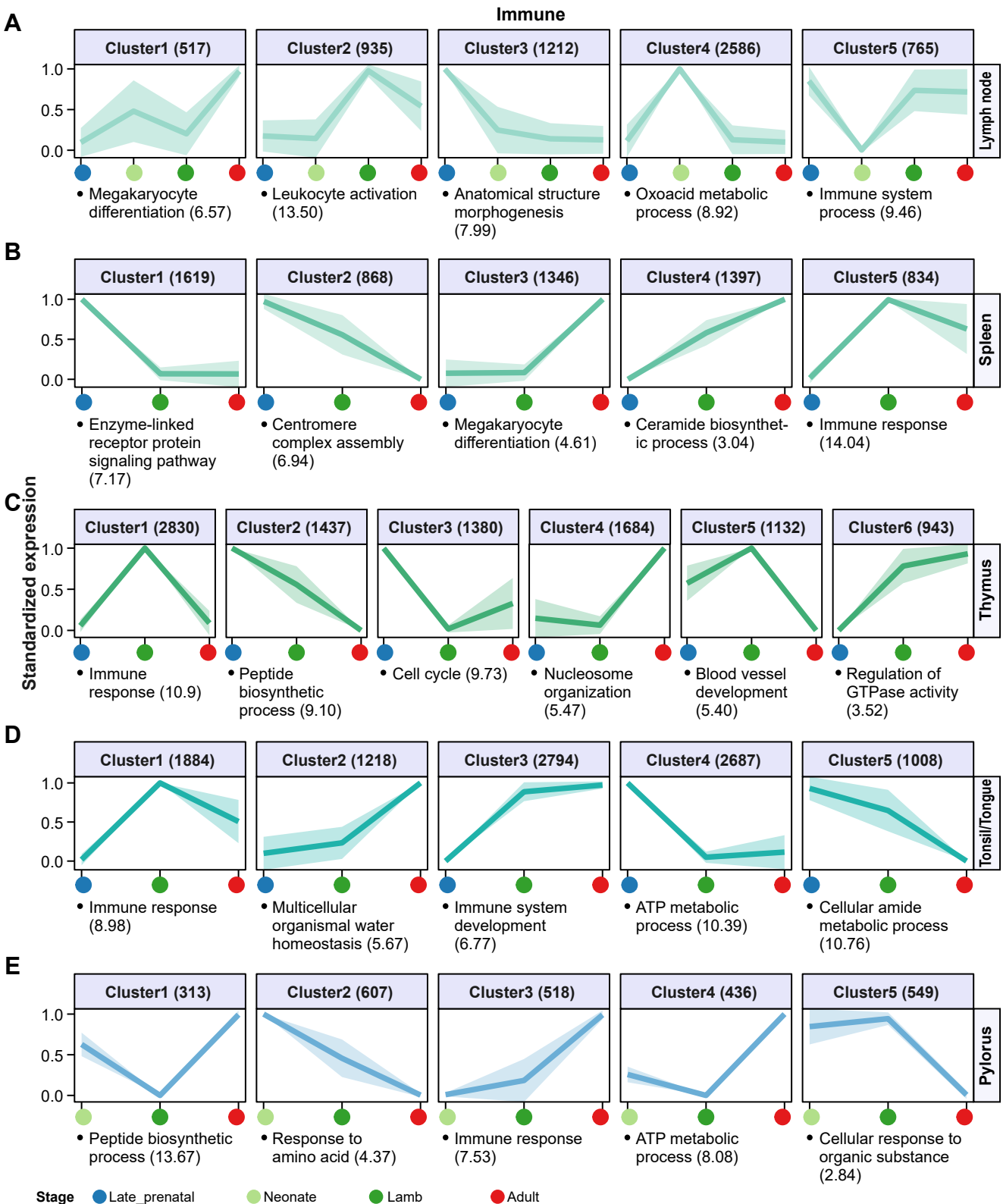

Supplement: qzaf020_Supplementary_Data [file qzaf020_supplementary_data.zip › Figure_S14.pdf]

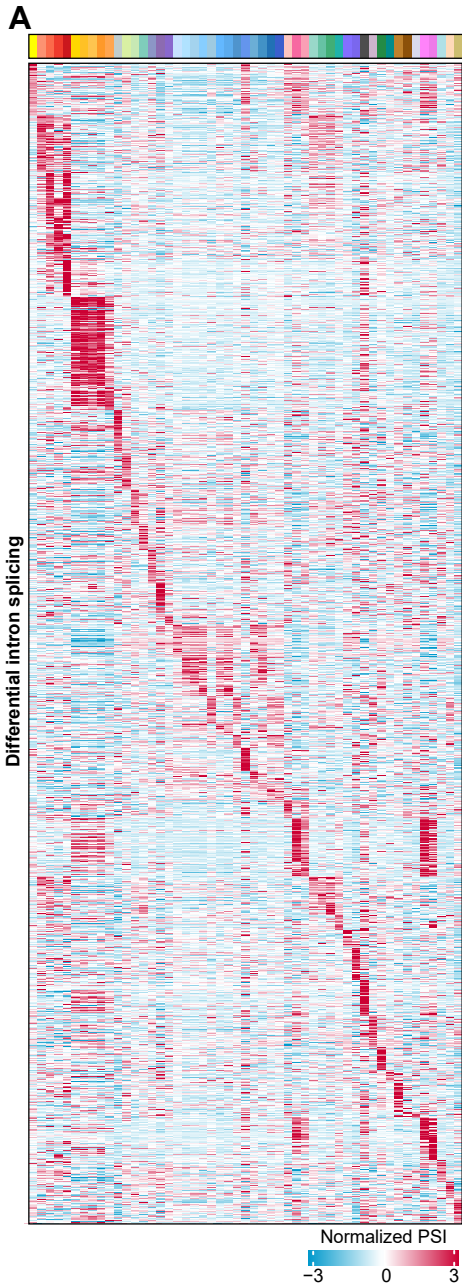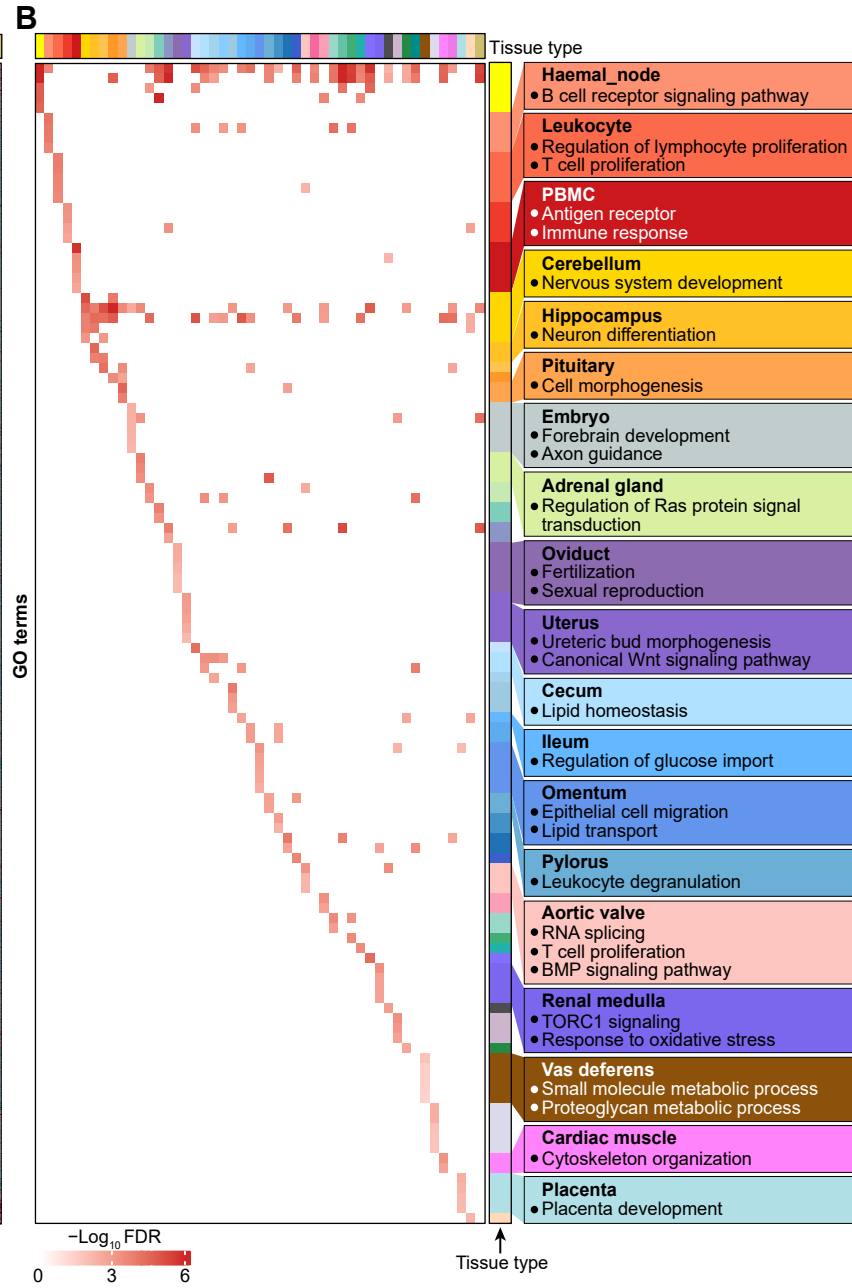

Supplement: qzaf020_Supplementary_Data [file qzaf020_supplementary_data.zip › Figure_S7.pdf]

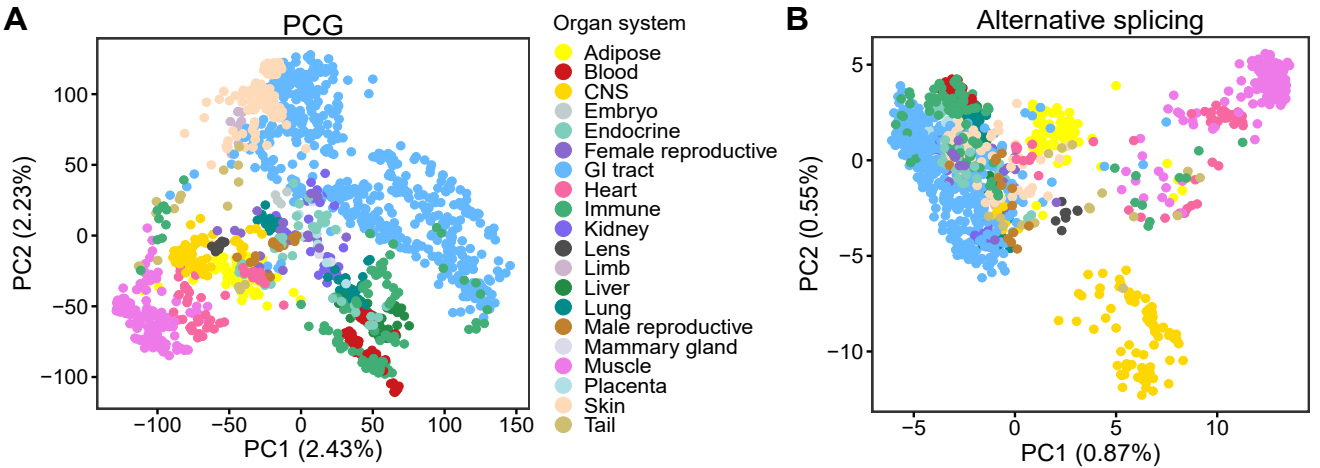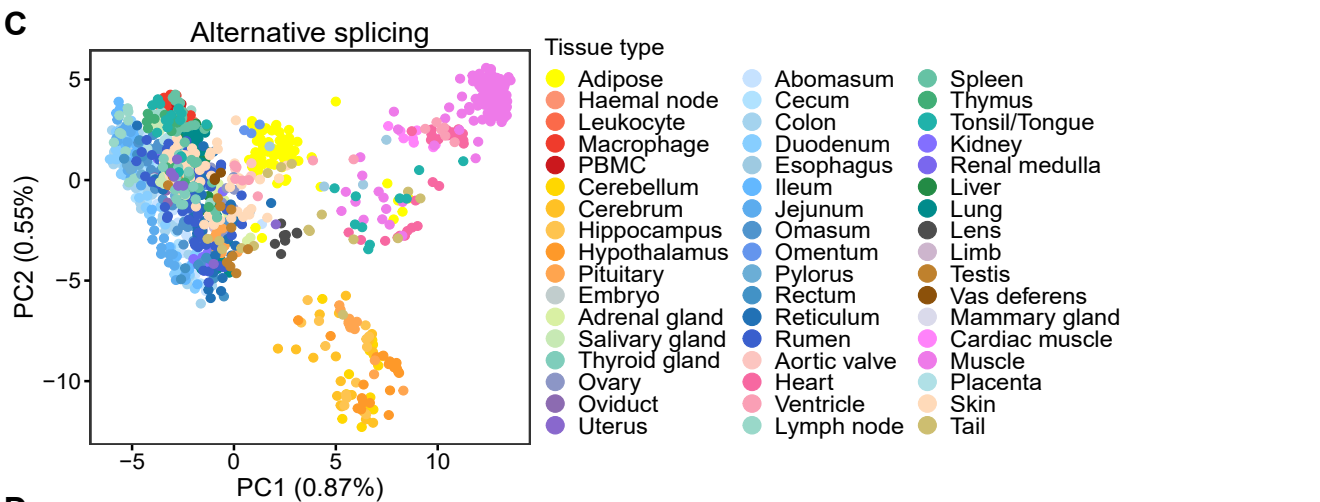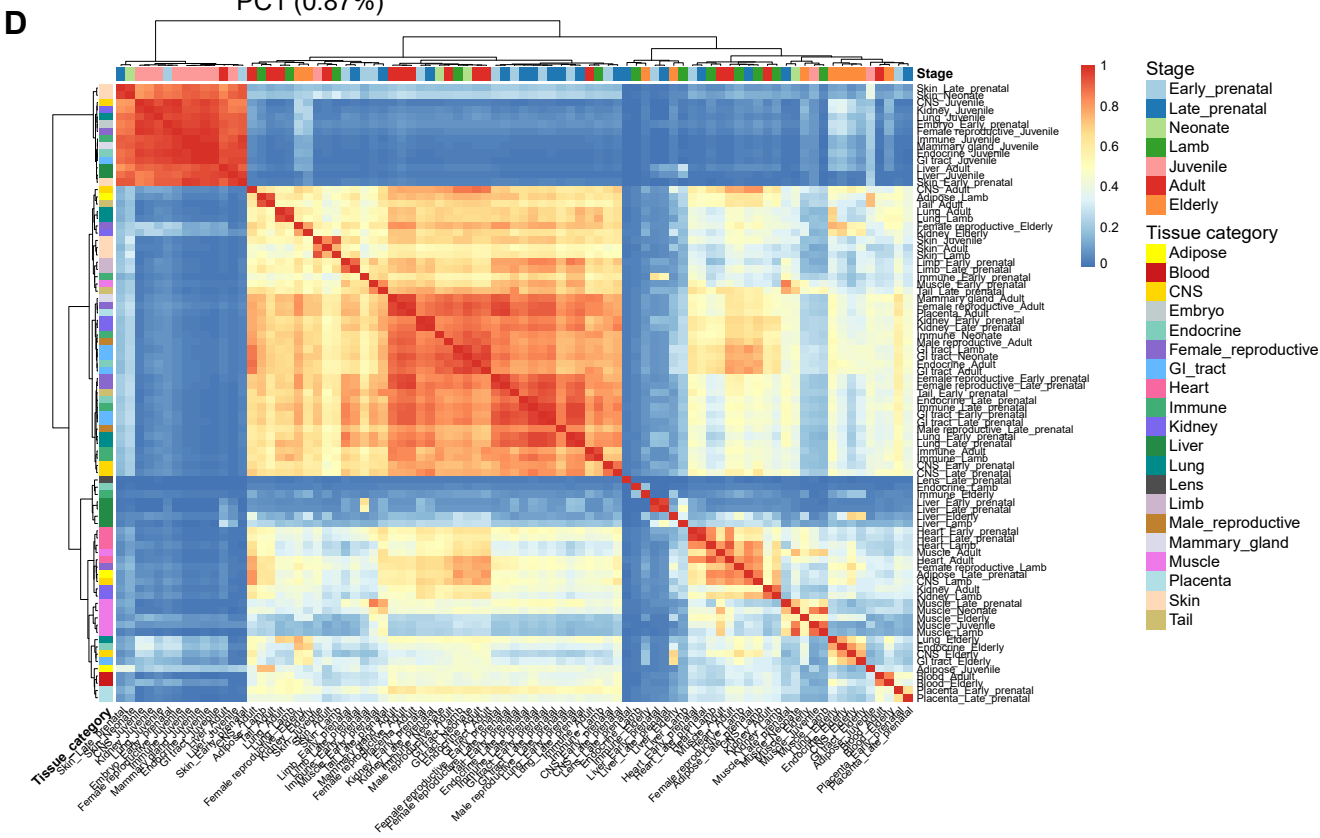

Supplement: qzaf020_Supplementary_Data [file qzaf020_supplementary_data.zip › Figure_S3.pdf]

**A****GI tract**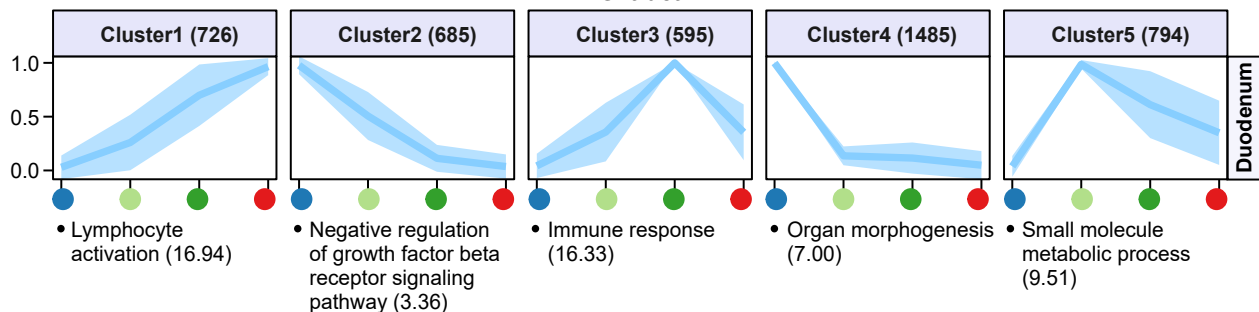**B**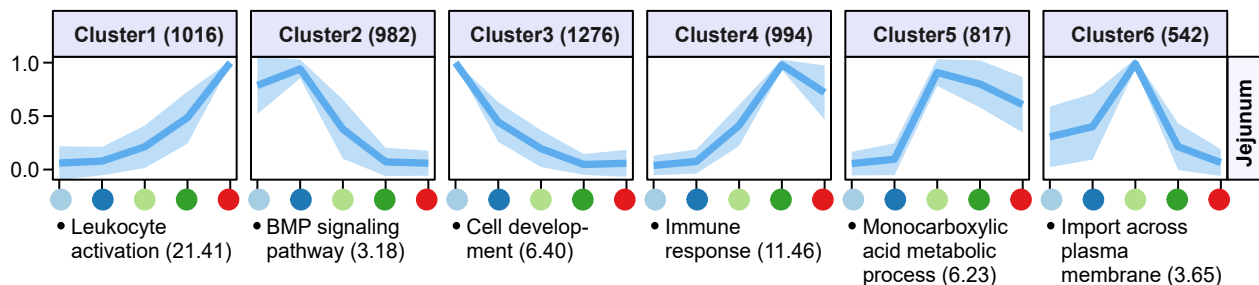**C**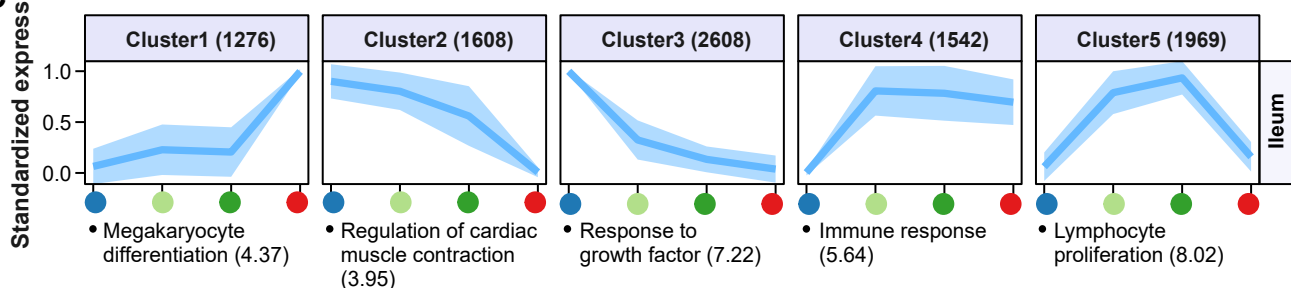**D**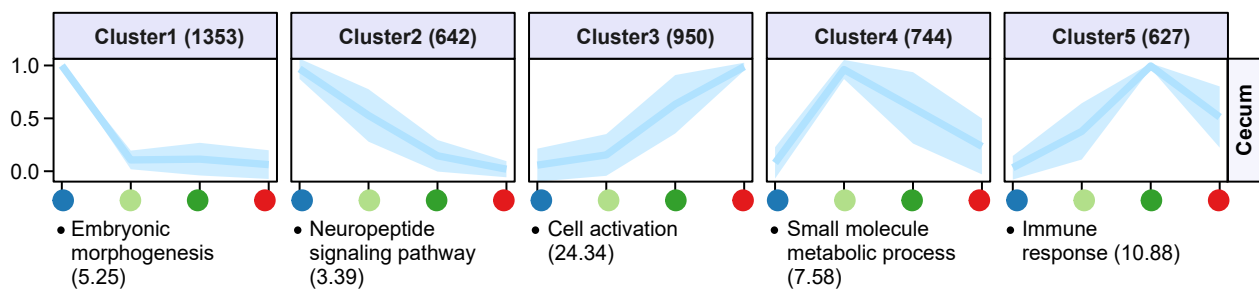**E**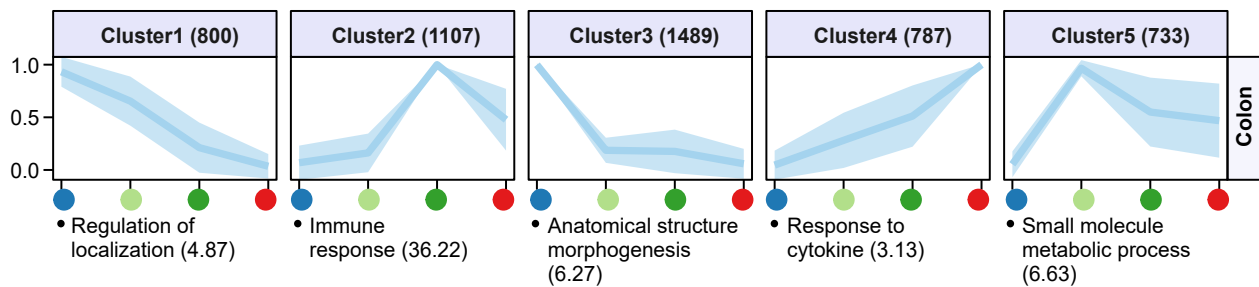

**Stage**    Early\_prenatal    Late\_prenatal    Neonate    Lamb    Adult

Supplement: qzaf020_Supplementary_Data [file qzaf020_supplementary_data.zip › Figure_S15.pdf]

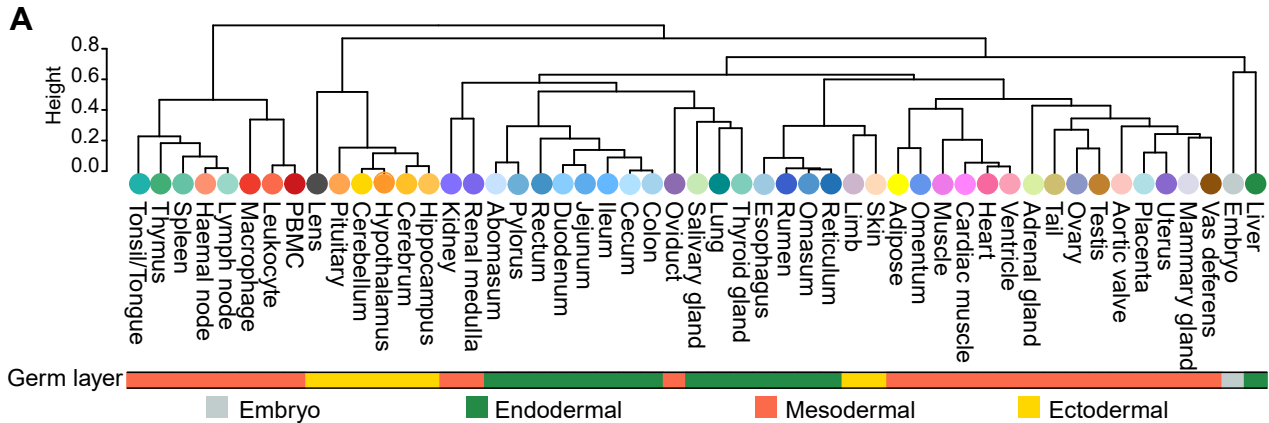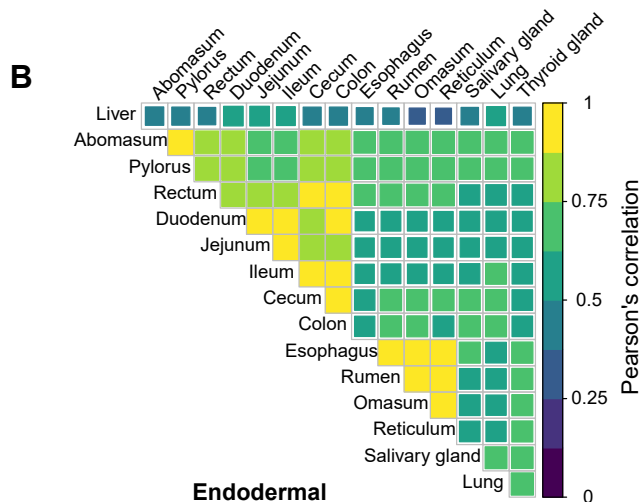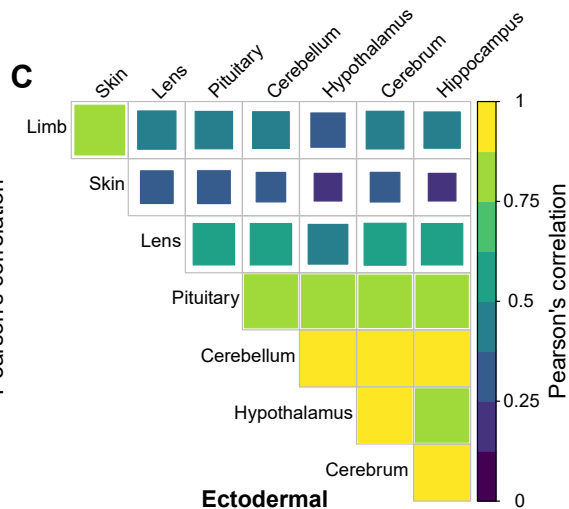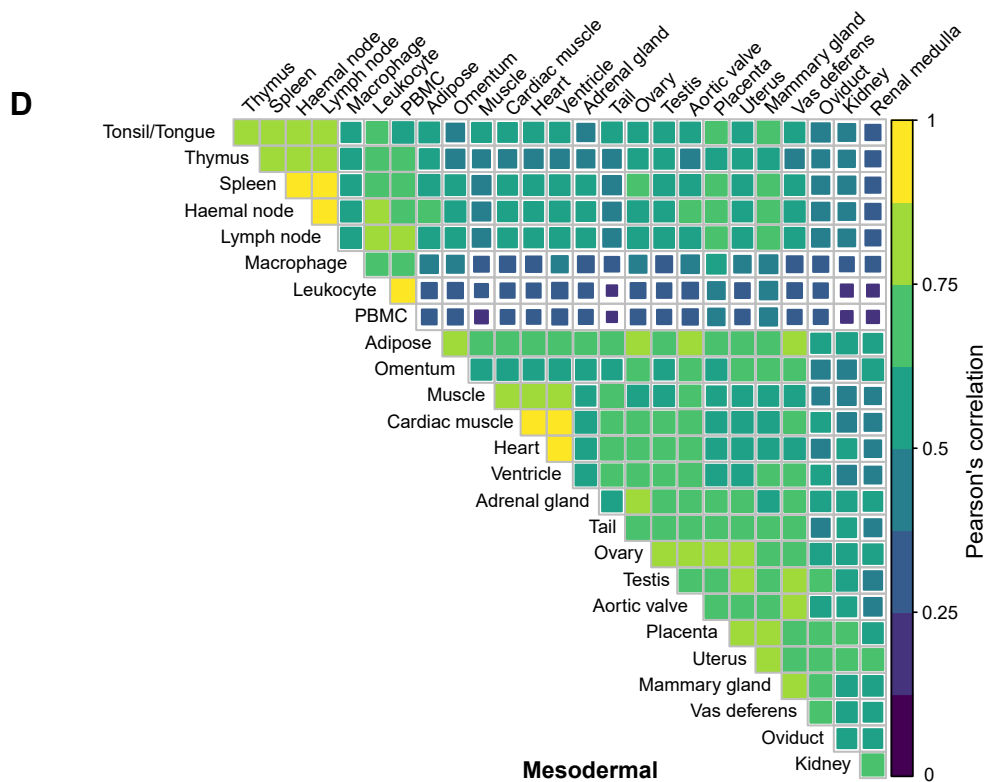

Supplement: qzaf020_Supplementary_Data [file qzaf020_supplementary_data.zip › Figure_S5.pdf]

# **A** Liver module-stage relationships

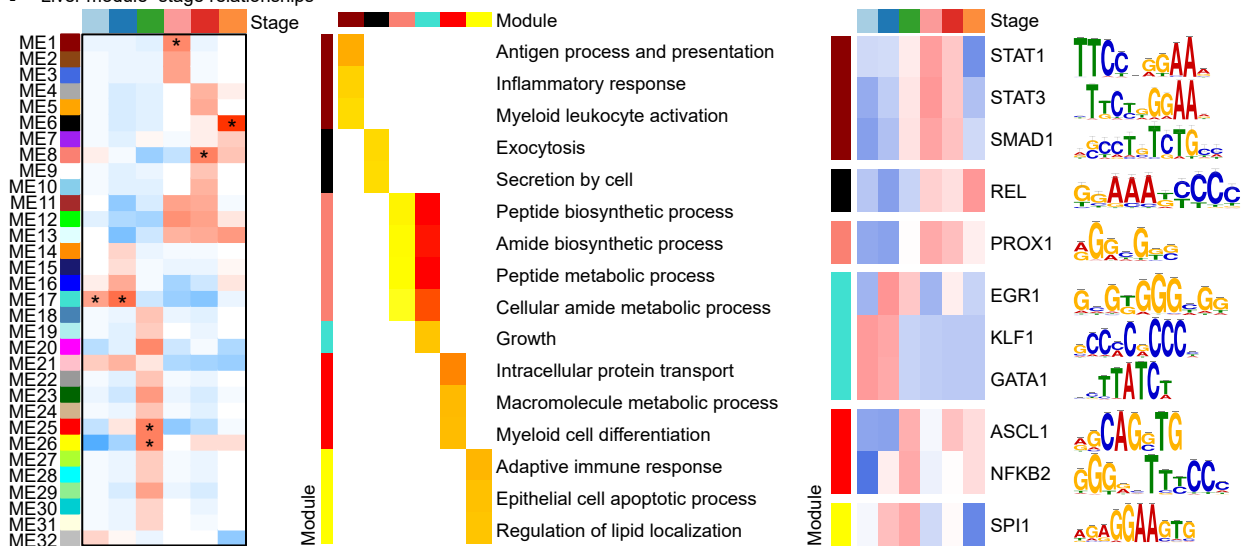

# **B** Kidney module-stage relationships

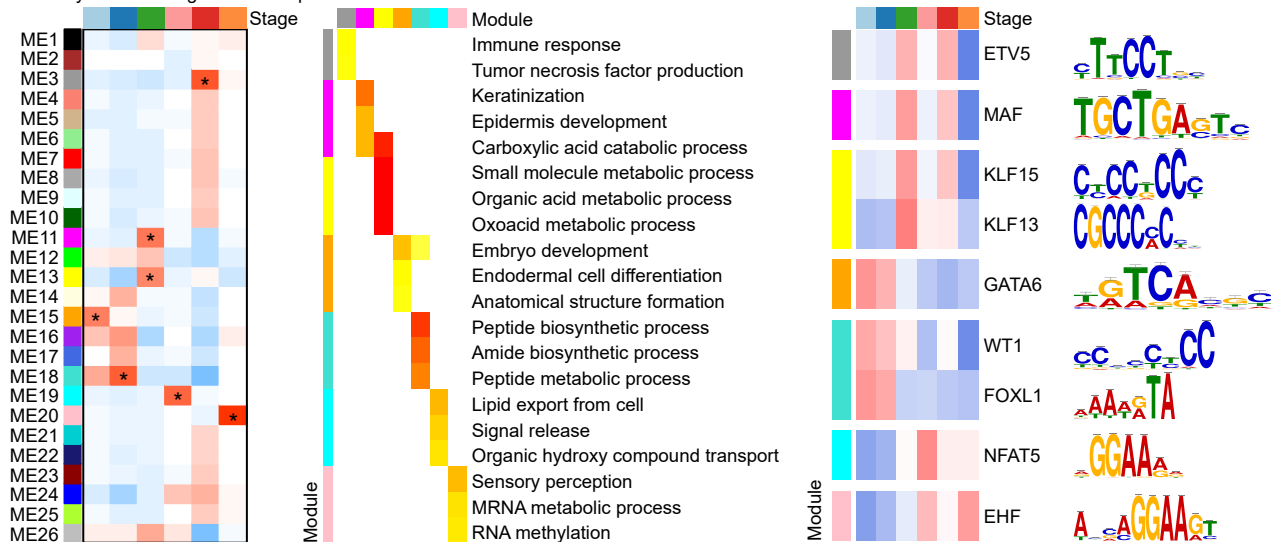

Supplement: qzaf020_Supplementary_Data [file qzaf020_supplementary_data.zip › Figure_S19.pdf]

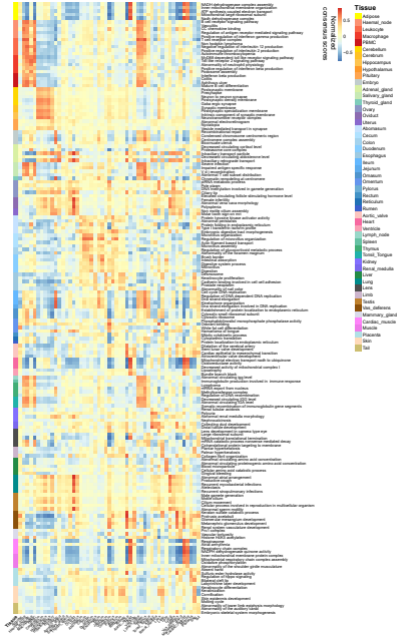

Supplement: qzaf020_Supplementary_Data [file qzaf020_supplementary_data.zip › Figure_S6.pdf]

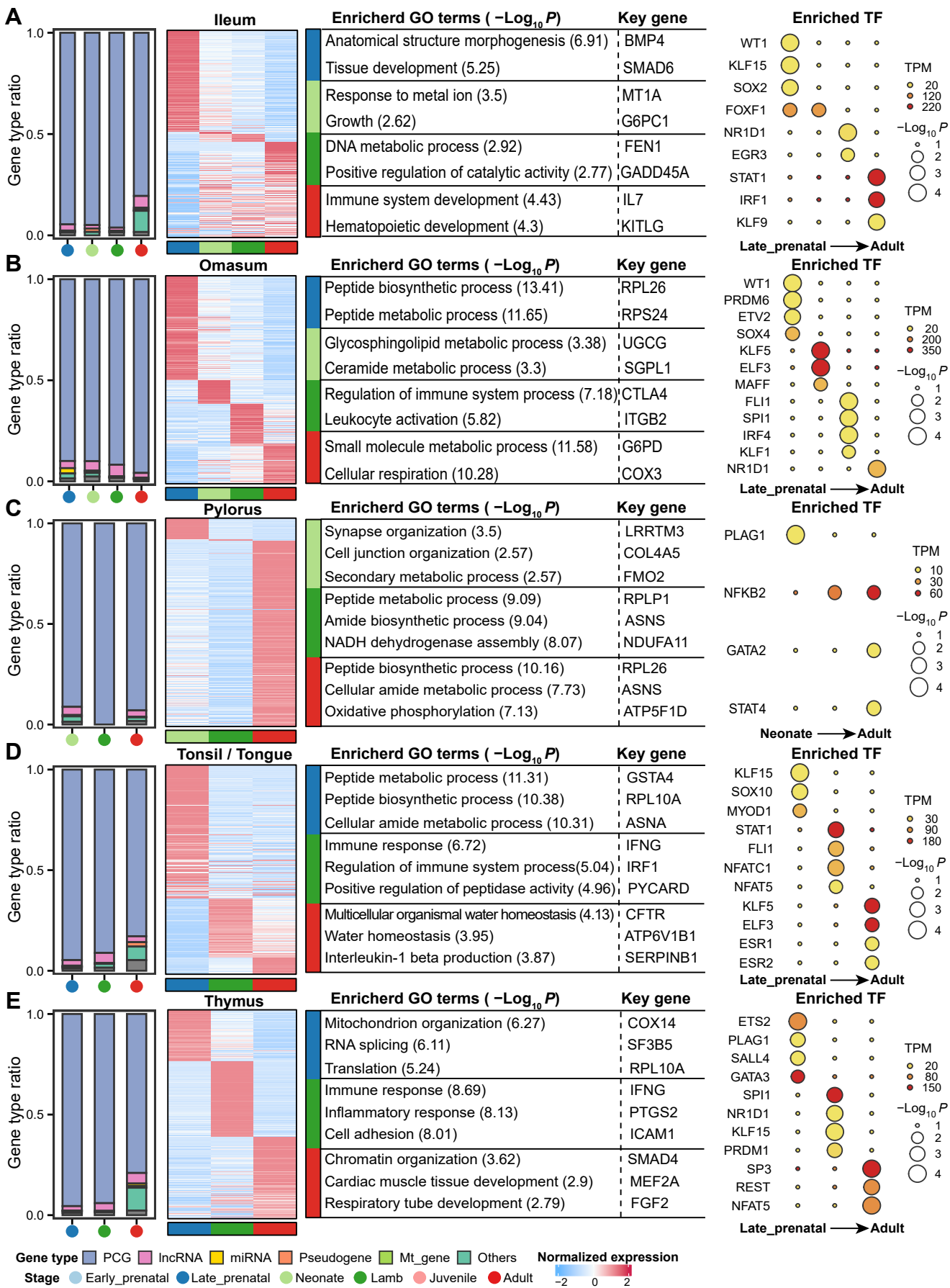

Supplement: qzaf020_Supplementary_Data [file qzaf020_supplementary_data.zip › Figure_S10.pdf]

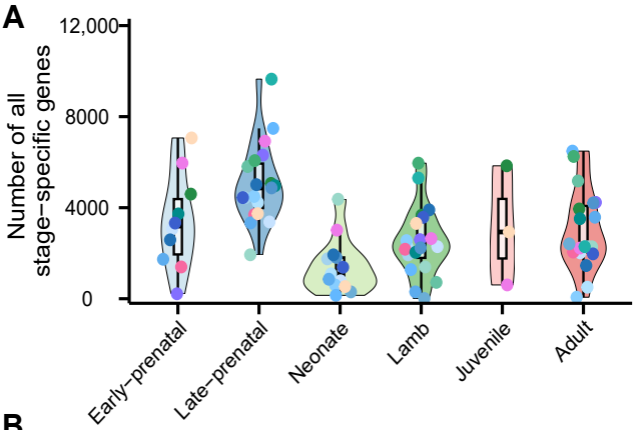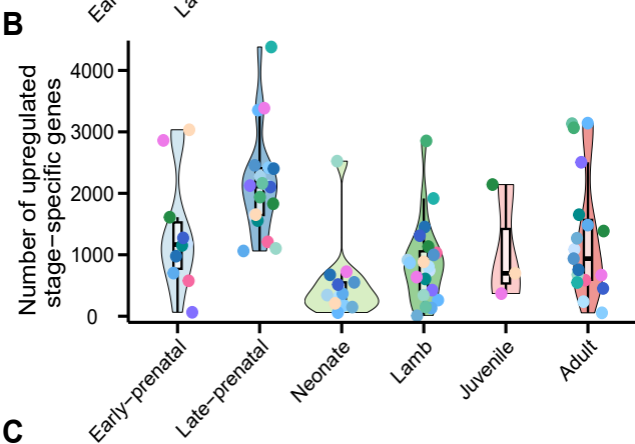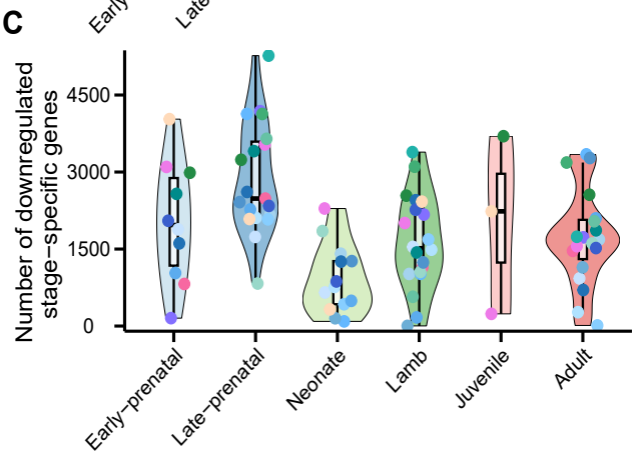

Supplement: qzaf020_Supplementary_Data [file qzaf020_supplementary_data.zip › Figure_S8.pdf]
